# Sonosensitive Cavitation Nuclei—A Customisable Platform Technology for Enhanced Therapeutic Delivery

**DOI:** 10.3390/molecules28237733

**Published:** 2023-11-23

**Authors:** Brian Lyons, Joel P. R. Balkaran, Darcy Dunn-Lawless, Veronica Lucian, Sara B. Keller, Colm S. O’Reilly, Luna Hu, Jeffrey Rubasingham, Malavika Nair, Robert Carlisle, Eleanor Stride, Michael Gray, Constantin Coussios

**Affiliations:** 1 Institute of Biomedical Engineering, Department of Engineering Science, University of Oxford, Oxford OX1 3PJ, UK; joel.balkaran@seh.ox.ac.uk (J.P.R.B.); darcy.dunn-lawless@magd.ox.ac.uk (D.D.-L.); veronica.lucian@st-hildas.ox.ac.uk (V.L.); sara.keller@eng.ox.ac.uk (S.B.K.); luna.hu@bnc.ox.ac.uk (L.H.); jeffrey.rubasingham@eng.ox.ac.uk (J.R.); malavika.nair@eng.ox.ac.uk (M.N.); robert.carlisle@eng.ox.ac.uk (R.C.); eleanor.stride@eng.ox.ac.uk (E.S.); michael.gray@eng.ox.ac.uk (M.G.); 2 Botnar Research Centre, Nuffield Department of Orthopaedics, Rheumatology and Musculoskeletal Sciences (NDORMS), University of Oxford, Oxford OX1 3PJ, UK; colm.oreilly@ndorms.ox.ac.uk

**Keywords:** drug delivery, ultrasound, cavitation, sonosensitive, nanoparticles, cavitation nuclei

## Abstract

Ultrasound-mediated cavitation shows great promise for improving targeted drug delivery across a range of clinical applications. Cavitation nuclei—sound-sensitive constructs that enhance cavitation activity at lower pressures—have become a powerful adjuvant to ultrasound-based treatments, and more recently emerged as a drug delivery vehicle in their own right. The unique combination of physical, biological, and chemical effects that occur around these structures, as well as their varied compositions and morphologies, make cavitation nuclei an attractive platform for creating delivery systems tuned to particular therapeutics. In this review, we describe the structure and function of cavitation nuclei, approaches to their functionalization and customization, various clinical applications, progress toward real-world translation, and future directions for the field.

## 1. Introduction

Our understanding of the biological mechanisms underpinning the spectrum of human disease continues to increase at a remarkable pace. Yet despite this, the number of new drugs approved (per billion USD of R&D spending, adjusted for inflation) has roughly halved every 9 years since 1950 [1]. As such, it is increasingly apparent that traditional drug discovery pipelines alone are not sufficient [2], and significant consideration must also be given to developing complementary strategies to ensure the effective delivery of drugs to their intended targets. Given their larger size, delivery limitations are especially relevant to newer biological therapeutics, including antibodies, oncolytic viruses, and gene therapy vectors, which, unless overcome, may ultimately limit their efficacy in a wider patient population. Recent clinical studies have further demonstrated that even delivering more therapeutic to a target site does not guarantee an improved patient outcome; rather, that bioavailability is a complex issue which also depends heavily on the mechanism by which the drug is delivered [3,4,5,6].

Stimuli-responsive drug delivery systems represent a potential solution to this problem as they are minimally invasive yet enable deep tissue penetration. While many stimuli can be utilized (e.g., light [7], heat [8], or magnetic fields [9]), this review will primarily focus on ultrasound-mediated drug delivery and its progress towards clinical translation. The ability of ultrasound to enhance therapeutic delivery is due primarily to a physical effect known as cavitation, defined here as the nucleation, oscillation, collapse, or other excitation of gas cavities (bubbles) in a fluid due to sound pressure changes [10]. While bubble behavior exists on a spectrum from extremely violent collapse to gentle pulsing, it is often classified into “inertial” and “non-inertial” cavitation. In broad terms, inertial cavitation is generally associated with higher pressures, unstable bubble growth, and violent collapse, while non-inertial cavitation generally occurs at lower pressures and involves lower-amplitude linear and non-linear oscillations about an equilibrium radius [11]. 

**Figure 1:**
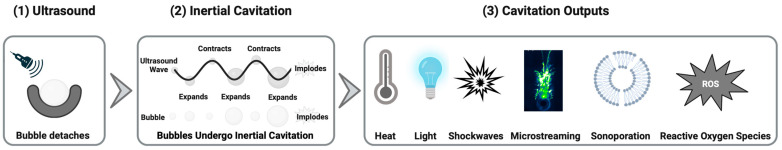
Therapeutic impact of cavitation.

Acoustic cavitation is relevant to a wide range of medical treatments as it can generate a diverse set of physical, chemical, and biological effects. Cavitating bubbles that oscillate repeatedly may induce “microstreaming”, a form of convectional fluid flow around themselves [12,13,14]. Where this flow occurs very close to cells, the high shear stresses produced may cause the cell membrane to reversibly or irreversibly open in places, in a process known as “sonoporation” [14,15]. Inertial cavitation in particular can also generate heat [16,17,18,19], light [16,17,18,19], shockwaves [20,21], and reactive oxygen species [22,23], and high-speed fluid “microjets” [11] (Figure 1). While the therapeutic impact of the above stimuli are frequently studied in isolation, ultrasound-induced cavitation represents a relatively unique opportunity to study their synergistic effect [24]. Crucially for precision drug delivery applications, these effects are highly localized around the cavitating bubbles, and can be targeted by directing the driving ultrasound source.

Cavitation can be initiated in tissue through the nucleation of bubbles from gas dissolved in the bodily fluids. However, this “endogenous” bubble formation is unpredictable and requires driving pressures high enough that the ultrasound alone may cause serious disruptions such as tissue boiling [25]. For enhancing drug delivery, the use of much lower pressures is preferable to avoid undesired, off-target, or uncontrolled bioeffects. This can be achieved by pre-seeding tissues with existing, stabilized gaseous cavities or favorable nucleation sites to promote bubble formation [26]. Such “cavitation nuclei” (CN) may therefore allow the broad therapeutic potential of cavitation and its various bioeffects to be unlocked at therapeutically safe ultrasound pressures.

**Figure 2:**
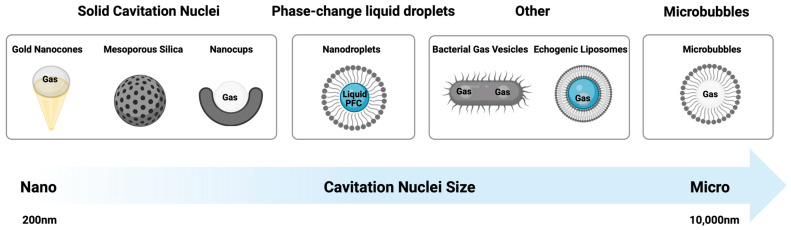
Types of cavitation nuclei.

CN are typically classified based on their physical make-up and related mechanism by which they entrap a stabilised gas bubble (Figure 2). Commonly used classifications include solid cavitation nuclei (e.g., polymeric nanocups, protein cavitation nuclei (pCaNs), gold nanocones, and mesoporous silica), gas-filled bubbles (with lipid, protein, or polymer shells), and phase-change liquid droplets, as well as emerging technologies, including echogenic liposomes and bacterial gas vesicles.

### 1.1. Gas-Filled Bubbles 

Gas-filled microbubbles are perhaps the most mature CN, having initially been developed as contrast agents for ultrasound imaging in the 1970s and 1980s [27]. Their size and structure afford microbubbles high compressibility and make them excellent scatterers of ultrasound waves, prompting their investigation in the context of medical imaging. However, when exposed to higher-intensity sound, these bubbles can also experience size and shape oscillation, collapse, reformation, and other cavitation-associated processes, making them an effective form of CN [28]. Microbubbles have therefore been increasingly studied as a therapeutic agent to enhance cavitation bioeffects like heating, mechanical debridement, and drug delivery [29].

Microbubbles are typically 1–10 μm in diameter [30] and consist of a high-molecular-weight gas core surrounded by a thin (1 nm–200 nm) stabilizing shell [31]. Hydrophobic gases such as sulphur hexafluoride or perfluorocarbons are commonly used in microbubbles as their relatively large molecular masses and low solubility in water limit the speed of bubble dissolution, permitting a longer lifetime in vivo [28,32]. These gas pockets are encapsulated in a supportive membrane to help stabilize them against coalescence or fragmentation, and further limit their rate of dissolution. The shell of a microbubble can be derived from a wide array of lipids, polymers, or proteins. Lipid shells (e.g., [33]) are currently the most widely used CN, such as in the clinically approved microbubble contrast agents SonoVue, Definity, and Sonazoid [34]. The lipid molecules are arranged in an ordered, tightly packed monolayer around the gas pocket, with their hydrophilic headgroups facing outward and hydrophobic tails pointing inward [35]. Since this layer is very thin (approximately 3 nm thick [31]) and the molecules are held in their monolayer by only comparatively weak van der Waals and Coulomb interactions [31,36], lipid shells are very compliant and provide minimal resistance to changes in bubble volume. Smaller “nanobubbles” (with diameters typically ranging from 100 to 800 nm) have also been reported in the literature [37,38]. However, a recent study by Myers et al. [39] was unable to demonstrate acoustic activity of bubbles in this size range, thus limiting their current utility in a drug delivery context. 

Protein shells were investigated early in the development of microbubbles [40]; Albunex, one of the first FDA-approved microbubble contrast agents [31], and its successor Optison both feature a shell of human serum albumin [41]. Protein microbubbles typically feature a thicker (15–30 nm [41]) wall than lipid bubbles, and are less compliant as the individual protein molecules are crosslinked by disulfide bonds [42]. This makes protein bubbles more resistant to dissolution, but depending on the thickness and degree of crosslinking in the shell, this construction may dampen their acoustic response and therefore inhibit cavitation activity. 

A wide variety of polymer-derived microbubbles (such as those made from poly (n-butyl cyanoacrylate) [43] now exist, with shell thicknesses ranging from 1 to 200 nm. Should it be desirable, this enables the generation of CN with shell rigidities far above what can typically be achieved for lipid or protein bubbles [44]. As such, these bubbles can be very stable in vivo, but their cavitation activity may be dampened to a greater degree than even protein bubbles. Instead of oscillating gently at lower pressures and gradually progressing to more intense inertial cavitation as pressure is increased (as is observed in many other bubble species), polymer bubbles instead respond minimally to low-amplitude sound and fracture violently under high pressures, allowing their entrapped gas to escape in a high-velocity jet [45]. To date, no polymer microbubbles have been approved for clinical use. 

Microbubbles are most commonly manufactured by emulsifying the desired shell material in an immiscible carrier fluid (typically aqueous) by either sonication [46] or high-shear stirring [47]. Sonication is often used to synthesize lipid and protein bubbles and involves the application of high-intensity ultrasound to emulsify the shell precursor (e.g., 22.5 kHz for 20 s [33]). Additionally, in the case of protein bubbles, cavitation induced by ultrasound generates reactive oxygen species which enhance the cysteine cross-linking reaction between molecules in the shell, increasing its rigidity [42,48]. High-shear emulsification produces microbubbles by stirring the shell precursor very rapidly (e.g., 5000 rpm for 5 min [49]), and is particularly used for polymer microbubbles [50]. These techniques offer a high yield but produce a broadly polydisperse population of bubbles, which has prompted the development of a raft of newer production methods, including microfluidics [51], membrane emulsification [52], layer-by-layer deposition [53], coaxial electrohydrodynamic atomization (CEHDA) [54], and inkjet printing [55]. These approaches generate controllable, monodisperse bubble populations, but are currently limited in terms of yield, production time, and other factors—see [50,56] for further details. 

In terms of safety, the majority of approved microbubbles contain non-atmospheric gases such as sulphur hexafluoride or octofluoropentane in extremely small quantities (<20 uL per 2 mL dose). The pharmacokinetic data provided as part of the regulatory filings for these microbubbles report that these gases are cleared by the lungs, with 100% of the administered dose being recovered 15 min after injection. 

### 1.2. Nanodroplets 

Nanometre-scale liquid droplets have garnered considerable interest as a form of CN in the last 20 years [11]. These droplets comprise a volatile liquid core (typically a perfluorocarbon) wrapped in a supportive shell [57], in a structure similar to microbubbles. Nanodroplets can be triggered with ultrasound to expand into a microbubble, a process known as acoustic droplet vaporization (ADV) [58]. The former droplets will then cavitate in a similar—but notably, not identical [59,60]—manner to standard microbubbles in response to ultrasound stimulation. 

The rationale behind this additional vaporization step is that droplets in their liquid form can survive longer in circulation than gas bubbles. Indeed, Keipert et al. demonstrated a half-life of 1–6 h in a rat model [61], depending on nanodroplet size, compared to 1–5 min reported for MB in mice [62]; should such an advantage also be seen in patients it would provide a compelling argument for nanodroplets. The core substance of a nanodroplet is typically a perfluorocarbon [63], and is selected to have a boiling point below body temperature (e.g., 29 °C for perfluoropentane [64]). Despite this, the droplet core is kept in a superheated liquid state in vivo by a combination of factors, including the Laplace pressure exerted on it by the surface tension of the surrounding fluid [65]. The perfluorocarbon remains in a meta-stable liquid state until this pressure is alleviated.for example, by the negative pressure half-cycle of an applied ultrasound wave. No longer constrained, the core rapidly boils and expands to several times its original diameter, e.g., from 416 nm to 8 μm [66]. Homogenous nucleation theory [65] is an alternate mechanism of droplet vaporization and may provide a more accurate explanation for the observed stability of superheated PFC nanodroplets. Droplet aggregation in biological liquids (e.g., serum) has also been shown to significantly impact the rate of vaporization [67].

Nanodroplets have been formulated with lipid [59,68,69,70,71,72], protein [73,74,75], and polymer [64,76,77,78] shells, similar in composition to microbubble walls. As in microbubbles, the shell acts to limit core efflux and reduce coalescence [74]. In nanodroplets, however, the shell also reduces the surface tension at the droplet surface, thereby reducing the Laplace pressure and helping to balance stability with ease of vaporization [65,79]. Droplet shells are thought to thin by around 25x when the core expands from liquid to gas [80], and are comparable to a thin microbubble shell when in vaporized form (e.g., 6 nm polymer shells in [78]). 

A number of the emulsification methods used to make microbubbles, including sonication, various forms of agitation, membrane emulsification, and microfluidics, can also be applied to manufacture nanodroplets—the primary difference being that the dispersed phase is a liquid instead of a gas [79]. Other methods of producing nanodroplets include microbubble condensation [59] and spontaneous nucleation [81]. The former technique involves exposing microbubbles to increased pressure and reduced temperature to condense the core substance into liquid form (e.g., for octafluoropropane, −10 °C for 2 min followed by 350 kPa pressurization [82]). As the core condenses, the shell wrinkles and can shed material as it has considerably less interface area to cover, then it re-expands upon later ADV [70]. Spontaneous nucleation involves dissolving the core and shell materials in a suitable solvent, then rapidly reducing the mixture’s solubility, such as by adding water [81]. This allows the shell and core substances to fall out of solution, forming a dispersion of nanodroplets.

### 1.3. Solid Cavitation Nuclei 

Solid cavitation nuclei are perhaps the most varied CN subgroup, particularly in terms of their morphological diversity, chemical composition, and size. However, they are unified by a deliberate design feature, namely the inclusion of hydrophobic cavities to allow entrapment of a stabilized gas bubble, thus providing a suitable nucleation site. Early examples of solid CN (such as the use of talcum powder) were based on observations that nano-sized air bubbles can readily become trapped on naturally occurring hydrophobic rough surfaces [83]. 

#### 1.3.1. Polymeric Nanocups

Ultrasound-activated polymeric nanocups were first described by Kwan et al. [84]. These sub-micron particles are composed of a polystyrene core with a crosslinked divinyl benzene and methacrylate shell which are ultimately deformed into a “cup-like” morphology. This indentation can provide a suitable surface for stabilizing an entrapped gas bubble on the surface of each particle, or act as a hydrophobic surface for facilitating nucleation. The diameter of a nanocup was shown to be readily tuneable by varying the size of the initial polystyrene seed particles.

This reduced size, with respect to microbubble CN, allows them to potentially travel further into microvasculature, which may give a delivery advantage. Due to their solid structure, they are also not destroyed by exposure to ultrasound, thus potentially allowing sustained cavitation to occur. However, a disadvantage of this same structural stability is that loading and releasing a drug from the core remains challenging. In addition, conjugation of a drug to the shell may disrupt the hydrophobic surface such that it may no longer entrap a gas bubble, which also limits its delivery capacity. Typically, polymeric nanocups also require a higher ultrasound pressure to activate than traditional lipid bubbles. 

Other reports of solid CN approaches include a paper by Su et al. on the development of multi-cavity PLGA microparticles which offer a more biodegradable variant of a solid CN [85]. A more recent development further proposes the use of a protein template obtained by solvent evaporation as a biodegradable single-cavity CN agent [86].

Solid gas-stabilizing CN are typically manufactured using minute quantities of atmospheric gases, most commonly air or oxygen, and therefore would not require further characterization in terms of pharmacokinetic gas clearance. 

#### 1.3.2. Gold Nanoparticles

Gold nanocones as a CN species were developed by Mannaris et al. as a modified synthesis of the non-cavitating gold nanoparticles described by Zhang et al. [87]. The particular formulation developed by Zhang was based off the violent vaporization of hexane trapped in hemispherical gold nanoparticles formed by ultrasonic sonication of a bi-phasic liquid–liquid mixture, which built off of Suslick’s [88,89,90,91] theory of rapid vaporization being capable of modifying particle surface morphology. To produce cavitating nanocones, the synthesis was modified to include a drying step, which allowed for the entrapping of bubbles upon resuspension in an aqueous medium, similar to the mechanism of bubble entrapment seen with nanocups. 

Subsequent acoustic characterization demonstrated inertial cavitation activity at both 0.5 and 1.6 MHz. The particles had an average hydrodynamic diameter of 200 nm (measured by DLS) and an internal cavity diameter of 150 nm by TEM, with an average concentration of 1.2 × 10^9^ particles per mL. Minimal biotoxicity was observed in vitro with the produced concentration of particles. At 0.5 MHz, it was found that a peak negative pressure of 2 MPa was needed to guarantee inertial cavitation, and for 1.6 MHz, this pressure rose to 3.5 MPa. Acoustic activation of the nanocones in agar flow channels demonstrated directional extravasation of fluorescent dye, highlighting the potential for targeting applications in drug delivery. Nevertheless, some challenges remain, including the tendency of these CN to aggregate, which can cause the entrapped bubbles to coalesce and increase the potential for off-target or uncontrolled bioeffects when acoustically excited [92].

Sazgarnia et al. [93] also investigated gold nanoparticles as an acoustic therapeutic by attempting to utilize the ability of solid particles in a fluid environment to act as a nucleation site for bubbles. They specifically exploited the tendency of gold particles to aggregate, which inherently increased the ease of inducing bubble formation. Their work involved the treatment of CT26 murine tumor models using pulsed laser light to encourage bubble nucleation before acoustically targeting the tumor. Of note is that they injected their gold nanoparticles intratumorally, thus ensuring the maximum local accumulation to reduce the bubble-forming threshold, as opposed to more typical administration routes like intravenous infusion, where most particles accumulate in the liver and spleen. Their study is limited, however, in that it did not monitor the cavitation activity produced, and thus their underlying hypothesis that light and acoustic stimulation of gold nanoparticles could promote an antitumor response is unverified. This area therefore remains untested in the application of drug delivery, but could show promise with further studies, given the frequent use of gold nanoparticles in therapies.

#### 1.3.3. Mesoporous Silica 

Advancements in the catalyst industry from the late 1980s onwards led to the development of mesoporous silica nanoparticles (MSNs) [94,95]. These were characterized by an ordered pore distribution, with homogeneous sizes in the range of 2 and 10 nm, a pore volume of approximately 1 cm^3^/g, and a surface area up to 1000 m^2^/g [96]. Being biodegradable, their physicochemical robustness and ease of functionalization through silanol chemistry was of immediate interest in a drug delivery context. In addition, their high surface area and associated pore volume suggested additional potential. However, despite intensive development and substantial further improvements to their drug-loading capacity, including the establishment of hollow core variants [97], a suitable in vivo targeting mechanism has ultimately limited their clinical translation. 

To address this, several groups [96,98,99,100] have since reported on the ability of MSNs to entrap stabilized gas bubbles, thus rendering them potentially ultrasound-responsive and providing a possible delivery mechanism. The first publication to establish this approach was Kim et al. [100], who encapsulated ibuprofen before using focused ultrasound-mediated cavitation to release it from the core. Paris et al. [98] demonstrated how focused ultrasound enhanced the extravasation of mesoporous silica nanoparticles using an in vitro flow-through agarose tissue phantom. Sviridov et al. have also described the use of MSN as a CN, but in the context of tissue heating rather than drug delivery [99]. Other examples are also provided by Lee et al., Milgroom et al., and Ma et al. [101,102,103]. 

In terms of synthesis, MSNs can now be made via a wide array of methods (including the original sol-gel process, microwave synthesis, hydrothermal synthesis, template synthesis, modified aerogel methods, soft and hard templating methods, and fast self-assembly), with each providing their own advantages/disadvantages [104].

### 1.4. Alternative Cavitation Nuclei

In recent years, some novel CN have been proposed which take the same basic form of a gas pocket wrapped in a stabilizing shell, but do not fit into the established categories of lipid monolayer, protein, or polymer microbubbles. These include echogenic liposomes [105] and bacterial gas vesicles [106]. Echogenic liposomes (also referred to as acoustically active liposomes or AALs) are believed to comprise a lipid bilayer shell around 1–2 µm in diameter, purportedly filled with a mix of gas and liquid [105]. These particles are designed to encapsulate comparatively large volumes of a drug until fractured by ultrasound exposure [107]. Bacterial gas vesicles are a fascinating addition to the cohort of CN: naturally occurring, cylindrical protein shells (2 nm wall thickness) filled with air that evolved in certain bacteria to allow them to float [108]. These vesicles can be encoded into a bacterial genome, genetically engineered to target certain cells by expressing different receptors on their shell, then mass-manufactured by simply culturing the bacteria [108]. However, their small size (45–250 nm wide, 100–600 nm long) may limit their cavitation activity [106]. Non-spherical “rod shaped” microbubbles have also been developed by Dasgupta et al. [109]. This unique morphology resulted in reduced phagocytosis, prolonged circulation time, enhanced margination, and enhanced blood-brain barrier (BBB) permeation. 

### 1.5. Summary

The wide variability of CN described above renders each subtype inherent advantages and disadvantages that may ultimately pre-dispose their suitability to a given therapeutic application. For example, some solid CN (such as gold or polymeric nanocups) are not biodegradable, but this interrelated structural stability may give them an enhanced in vivo circulation time and increased cavitation duration [84]. In contrast, lipid- and protein-based microbubbles are fully biodegradable but tend to be bigger compared to some solid CN, limiting their access to the microvasculature. Solid CN and nanodroplets typically also cannot be imaged before activation, which is a key advantage of microbubbles [11].

Mesoporous silica particles have a much higher therapeutic loading capacity [110] than most other CN, but their usage-specific release mechanisms need to be determined. Nanodroplets have some unique size advantages, but their stability in a clinical setting remains untested. The key to developing an optimal delivery strategy is to prioritize the most important application-specific performance criteria early in the process, so that the correct CN can be selected. 

## 2. Composition Optimization of Cavitation Nuclei for Enhanced Delivery of Therapeutics

**Figure 3:**
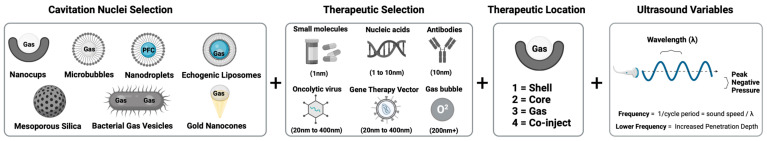
Cavitation nuclei therapeutic variables for enhancing delivery of therapeutics.

Once a suitable combination of CN and therapeutic has been selected, a range of additional variables can be optimized for further impact, with a primary consideration being the location of a chosen therapeutic (Figure 3). 

### 2.1. Co-Injection

In the simplest iteration, any of the above CN can be co-administered simultaneously with unmodified therapeutics by intravenous injection or infusion, and subsequent ultrasound exposure. A key advantage of this approach is that it is readily compatible with all commercially approved therapeutics, without the need for additional re-formulation, with mixing of the two species occurring in the bloodstream. However, for enhanced delivery to ultimately occur, it relies on the assumption that sufficient quantities of the CN and the therapeutic will be co-located at the targeted area when cavitational microstreaming is induced. 

Several pre-clinical studies have used co-administered cavitation nuclei to significantly enhance the delivery of co-administrated small molecules [111,112,113,114], antibodies, viral vectors [115,116,117,118], and nucleic acids [119,120,121]. There have also been several clinical trials evidencing the successful use of gas-filled microbubbles particles (such as Sonovue^®^, Optison^®^, Definity^®^) which have been approved as diagnostic ultrasound contrast agents to enhance the effects of standard chemotherapy in the context of chemotherapy for pancreatic cancer [122,123] or to treat pathologies that lie beyond the blood–brain barrier [124,125]. Most recently, a new class of solid gas-stabilizing CN (OxSonics SonoTran particles) were cleared by the UK’s Medicines and Health Regulatory Authority (MHRA) to enter first-in-human clinical trials (NCT02181075) for the enhancement of the delivery of co-administered small-molecule chemotherapeutics and antibodies to treat metastatic colorectal cancer in the liver. 

### 2.2. Shell

Several CN either have naturally occurring and directly targetable functional groups on their surface, or can be readily modified to incorporate them, thus allowing a wide array of additional therapeutics to be conjugated [11]. While the specifics may vary depending on the composition of the chosen CN, some common approaches can be considered. For lipid microbubbles and droplets, this is typically achieved by the addition of functionalized lipids prior to synthesis [31]. Common examples include lipids with groups such as DBCO, biotin, amines, or thiols, which all allow further conjugation via a range of well-established chemical reactions [126,127]. Some hydrophobic drugs can be directly incorporated into the lipid shell [128] and nucleic acids can be attached via electrostatic attraction, although there are questions over whether this approach would provide sufficient stability in humans [129]. There are also many examples of additional nanoparticles (such as liposomes and magnetic iron oxide particles) being directly incorporated into the lipid shell [130,131].

Protein-based microbubbles typically have a range of amino acids that can serve as naturally occurring conjugation sites. These include lysine (amine groups) and aspartic/glutamic acids (carboxylic acids), with some proteins also having accessible cysteine residues (thiols). In addition, these residues can be further modified for click chemistry or biotin/avidin binding sites via a wide array of commercially available heterobifunctional linkers [132]. Therapeutically relevant peptides or proteins can also be incorporated by adding them to the aqueous layer containing the primary shell protein (often albumin) prior to formation [133]. Due to the structural and chemical modification associated with the nanoparticle synthesis, questions may remain as to what degree of protein functionality and or binding ability is retained, but examples of functionality retention (using haemoglobin nanoparticles) exist [133].

Mesoporous silica CN can be readily loaded with a wide array of hydrophobic and hydrophilic therapeutics as a result of their characteristic surface pores [110]. A common variation on this approach is to subsequently block the pores with a stimuli-responsive polymer gatekeeper, thus tailoring drug release towards specific applications [110]. Silane chemistry can also be used to conjugate therapeutics to surface silanol groups on the external particle walls [134,135].

### 2.3. Core

Therapeutics can also be loaded inside the core of some CN. This has the advantage of protecting the cargo from unwanted degradation while in transit, but the mechanism of release and associated kinetics must be carefully considered. For incorporation into a CN core, there are several mechanisms of release that could be utilized including enzymatic degradation, pH, temperature, light and ultrasound [136].

In the context of lipid-based microbubbles, drugs can be loaded directly into the core by incorporating them into the oil layer frequently used to stabilize these nanoparticles [137]. Lipid derived nanodroplets can also be loaded with drugs in a similar manner [58]. In both cases the targeted release mechanism is typically ultrasound-induced cavitation, as this readily disrupts the lipid shell and releases any therapeutic from the core [58,137]. Mesoporous silica nanoparticles can be efficiently loaded with a high concentration of therapeutic via the addition of a post-grafted polymeric gatekeeper to seal the cargo inside [110]. Release mechanisms can be tuned by careful selection of the gate-keeper composition.

### 2.4. Gas

The choice of gas incorporated into a CN can also be viewed in a therapeutic context. For example, tumor hypoxia is a known barrier to effective cancer treatment and the ability to deliver oxygen bubbles could have a substantial therapeutic impact [138]. In addition, localized delivery of nitrous oxide could be of great benefit in a thrombolysis/cardiac environment. The use of CN in these contexts has been demonstrated in animal models [139,140], suggesting the potential, but as yet no clinical application has been approved.

In conclusion, the choice of therapeutic loading location will ultimately depend on its specific mode of action and intended interaction with cells. This should be given strong consideration as part of defining an appropriate delivery strategy. 

## 3. Key Ultrasound Considerations for Drug Delivery 

### 3.1. Ultrasound Parameters

**Figure 4:**
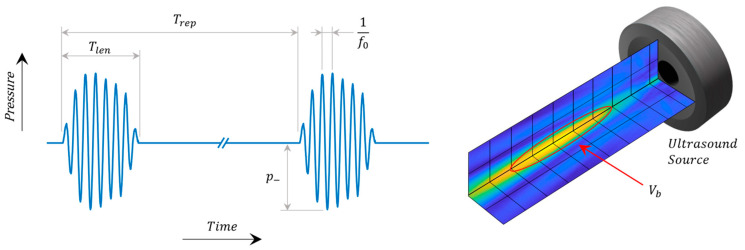
Key ultrasound parameters for drug delivery.

In addition to the CN and associated choice/location of a therapeutic, the applied US field can in principle be optimized to tune the cavitation activity for a given application. Common US specifications (Figure 4) include peak negative pressure ($p_{-}$), fundamental frequency ($f_{0}$), pulse length ($T_{len}$), pulse repetition period ($T_{rep}$), and main lobe beam volume ($V_{b}$). Together these describe the strength, time scale, and spatial scale of the pressure field. In very general terms, the strength of cavitation activity (oscillation amplitude, bubble growth, likelihood of inertial collapse) is increased by raising the magnitude of $p_{-}\;$(increases exterior tensile force), lowering $f_{0}$ (increases absolute time per cycle over which the force is applied), and increasing $T_{len}$ and shortening $T_{rep}$ (increases growth by rectified diffusion and heat deposition). Increasing $V_{b}$ enlarges the region over which the pressure and time scale factors act, and for example may expose a greater number of CN and increase their cumulative activity. 

Other common US parameters include those reported on diagnostic ultrasound equipment for safety monitoring [141]. The mechanical index, $MI=p_{-,0.3}\left(z\right)/\left(C_{MI}\;f^{1/2}\right)$, describes the risk of damage due to bubble collapse, where $p_{-,0.3}\left(z\right)$ is the pressure after derating assuming 0.3 dB/cm/MHz attenuation at depth $\left(z\right)$, and $C_{MI}$ is a unit correction constant. This index was developed specifically to assess risks with short diagnostic pulses (<5 cycles, or <10 μs) and duty cycles <1% to obviate rectified diffusion [142]. It is not meaningful to use MI as a safety guide when extrinsic CN are present, duty cycle >>1%, or pulses exceeding diagnostic length are used. 

Field descriptions in terms of intensity or power are also used in both diagnostic and therapeutic contexts, particularly when considering heating effects purely from the US in the absence of CN. Intensity and power metrics by themselves do not directly define the rarefactional pressure field, so they should be accompanied by the pressure descriptors listed above whenever possible.

**Figure 5:**
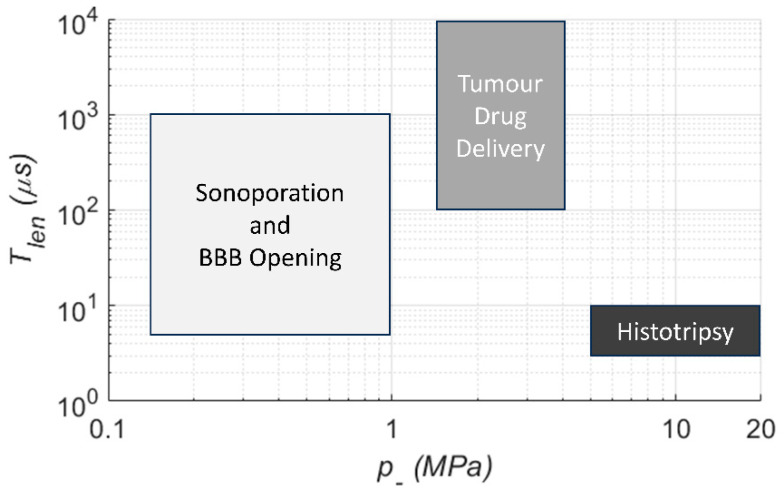
Generalized US parameter ranges by CN application.

Optimal US parameter ranges are strongly dependent on the choice of CN and its application (Figure 5). A comparative in vitro study with microbubbles, solid gas-entrapping particles, and droplets [69] showed that longer pulses (1000+ cycles) enhanced extravasation of a model drug when $p_{-}$ was chosen to ensure inertial cavitation. These kinds of settings have been carried forward into preclinical [116,143,144] and clinical drug delivery studies [145]. A further theme for in vivo work has been to place a floor on $T_{rep}$ (>1 s) to allow vascular replenishment of CN in the target region, whilst maintaining millisecond-scale $T_{len}$ [146]. Alternative schemes have also been investigated wherein a series of short (<5 μs) US bursts are employed for BBB opening [147]. 

In another comparative study with microbubbles and solid gas-entrapping particles for histotripsy [148], both CN substantially reduced the required $p_{-}$ relative to the intrinsic threshold, and while pulse lengths were kept at <4 μs, lengthening $T_{rep}$ from 0.1 to 2.0 s was seen to give enhanced control of bubble cloud shape. Aside from the differences in pulse timescales, the above literature shows trends of $p_{-}$ being smallest for BBB opening and reversible sonoporation (avoiding inertial cavitation), increasing for drug transport (inertial cavitation below tissue damage threshold), and highest for histotripsy, even when CN are introduced. 

Given the broad parameter space available, rather than relying solely on the literature values, it is best to optimize exposure conditions based on the combination of CN, the desired bioeffect, safety considerations, and the environment in which the CN are used. 

### 3.2. Cavitation Monitoring

Real-time guidance and safety assessment during US + CN treatment typically involves monitoring of ultrasound scattered or radiated by bubbles. Observations of scattered fields are facilitated by specific imaging transmissions that are temporally separated from the therapy pulse and allow examination of the inter-pulse bubble population [149]. Aside from potentially missing activity during the application of the therapy pulse, a critical limitation of these ‘active’ methods arises when using solid CN, which may be anechoic under imaging pulse conditions. 

‘Passive’ methods [150,151] form images based on bubble-radiated sound during application of the therapy pulse, and therefore capture bubble activity without restrictions on timing or pulse sequencing. However, passive methodological reliance on received bandwidth and receiver aperture commonly result in axial spatial resolution inferior to what is attainable with ‘active’ methods. This resolution gap has been narrowed through progress on advanced beamforming algorithms [152,153]. Recently, some hybrid monitoring methods [154,155] have been developed wherein short therapy pulses allow the use of causality constraints to significantly decrease bubble location uncertainty. 

Just as was seen for CN and US parameter choices, the selection of cavitation monitoring method has also varied widely by application. For clinical BBB opening with microbubbles and an MRI-guided hemispherical array [146], subharmonic emission levels have been transcranially monitored using a distributed array of receivers. These levels are used to control the therapy beam amplitude with the aim of minimizing the potential risk of tissue damage due to inertial cavitation while maximizing the likelihood of targeted BBB opening. For intratumoral drug delivery, broadband emissions in a frequency range well above $f_{0}$ have been imaged with a single array [144] or a pair [156] of arrays. Intrinsic threshold histotripsy monitoring employs a sparse array of receive-capable elements of the transmit array to observe bubble nucleation, collapse, and their relative timing [157]. 

All these methods are intended to provide essential physical insights into the therapy delivery process, although clinical evidence of the relationship between monitored/imaged cavitation activity, safety, and desired bioeffects is still being gathered.

## 4. Therapeutic Applications

**Figure 6:**
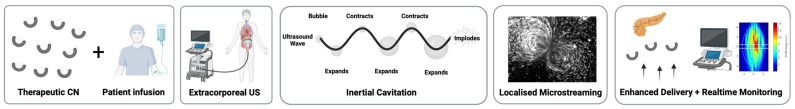
Overview of a potential sonosensitive cavitation nuclei delivery system.

The use of ultrasound as a sonosensitive cavitation nuclei delivery system has widespread potential across the whole spectrum of human disease (Figure 6). 

### 4.1. Drug Delivery to Solid Tumors 

Ninety percent of drugs under development fail after advancing to the early phases of clinical trials, with over half of them failing due to lack of efficacy [158]. Almost all the anti-cancer agents reaching the human trial phase would have had favorable preclinical data, reflecting the complex barriers to drug efficacy encountered in clinical translation [159]. Whilst a lack of efficacy in early-phase oncology trials could be multifactorial, there is strong evidence to suggest that suboptimal drug delivery and poor penetration of drugs in solid tumors contributes significantly to the reduced efficacy of anti-cancer agents [160,161]. Attempts to optimize drug delivery to the tumor by means of increasing the systemic dose of cytotoxic agents would invariably result in dose-limiting toxicities in the patient due to such agents targeting both healthy cells as well as target tumor cells indiscriminately. 

The last two decades have seen an exponential growth in the discovery of targeted drugs, designed to target the cancer cells with high potency whilst sparing normal cells [162]. Targeted therapies could be broadly classified into two categories based on their size: small molecules (mass <1 kDa, effective size <5 nm) and larger macromolecules such as monoclonal antibodies (mAbs) (100–150 kDa, ~10 nm) [11,163]. Many small molecules in targeted therapy are kinase inhibitors (in particular, tyrosine kinase inhibitors—TKIs) which block signalling pathways dysregulated during tumor formation. 

mABs specifically act on extracellular proteins as they are typically too large to enter the cells and so they inhibit tumor growth by preventing the interactions between receptors and ligands and triggering events such as antibody-directed cell cytotoxicity (ADCC) and complement-dependent cytotoxicity (CDC) [163]. Whilst the advances in targeted therapy have produced a paradigm shift in the survival outcomes of a subset of cancer patient populations, such as patients with non-small-cell lung cancer and targetable mutations [164], for some others the benefits have been modest at best [165]. A recent analysis has disappointingly highlighted that of the 207 cancer drugs approved by the FDA between the years 2016 and 2021, only 28 (14%) managed to displace the existing first-line therapies [166]. Failure of these highly specific agents to alter the landscape of current therapy emphasizes the importance and prescience of addressing the issue of suboptimal drug delivery and penetrance within the tumor. 

#### Clinical Progress 

Despite the breadth of preclinical data on ultrasound-enhanced drug delivery, clinical trials on ultrasound-enhanced drug delivery for extracranial solid tumors with published reported outcomes are limited. Several studies have looked at combining thermosensitive liposomes with ultrasound, as reviewed in Chaudhry et al. [167]. This includes TARDOX, a first-in-human clinical study looking at ultrasound-triggered targeted drug delivery of doxorubicin from thermosensitive liposomes in liver tumors. The study concluded that the approach is a safe and feasible delivery system and is also able to achieve single-cycle chemo-ablative response in liver tumors which are refractory to standard therapy [168]. Another clinical study utilized microbubbles and ultrasound for the enhancement of the delivery of chemotherapy to pancreatic tumors. Ten patients with inoperable pancreatic cancer were treated with gemcitabine chemotherapy followed by immediate intermittent bolus injections of SonoVue microbubbles and sonoporation using a diagnostic ultrasound machine. The authors reported an improvement in median survival of 8.7 months. However, it should be noted that the study did not have a control group and the results were compared to a historical control, requiring cautious interpretation [122]. SONCHIMIO, a randomized early-phase study in patients with metastatic colorectal cancer, is examining standard chemotherapy versus chemotherapy with sonoporation using microbubbles [169]. The trial has now completed recruitment after enrolling seven participants, but the results have not been published yet. Table 1 shows a summary of clinical trials on ultrasound-enhanced drug delivery in extra-cranial solid tumors, recruiting as of 12 January 2023. The table highlights that whilst consensus is emerging around the most appropriate target indications, with liver resident and pancreatic tumors commonly chosen, there is no agreement yet on the need for or type of CN.

### 4.2. Transdermal Vaccine Delivery

Needle-free administration of drugs through the skin has long been desired in clinical medicine, as it offers the potential for painless, non-invasive delivery which may avoid first-pass metabolism, and in the context of vaccination could provide greater activity per dose [179]. The primary obstacle to transcutaneous or transdermal drug delivery is the highly effective barrier of the stratum corneum (“SC”, the skin’s outermost layer), which excludes all but very small (<500 Da), lipophilic (log P = 1–3) therapeutic molecules from diffusing through to the body [180]. Ultrasound has been studied with increasing interest over the past 30 years as a method of overcoming the SC and delivering drugs through the skin, a process termed “sonophoresis” [181]. 

Of the many bioeffects of ultrasound, inertial cavitation has been repeatedly indicated as the primary mechanism behind sonophoresis [182,183,184,185]. The violent collapse of bubbles aids transport of drugs through the skin in two main ways: increasing the permeability of the SC and providing a convective force to “pump” drugs through it. Cavitation permeabilizes the skin primarily through high-velocity fluid microjets caused by asymmetric bubble collapse [183,185], which mechanically disrupt the SC and cause the reversible formation of channels through it. Microstreaming flows generated around cavitating bubbles can also help to push drug molecules through the skin, further improving delivery [186]. CN therefore hold significant potential to improve sonophoretic transcutaneous and transdermal vaccine and drug administration, as they allow much greater cavitation activity at much lower pressures than is possible with endogenous tissue nuclei alone.

Another promising application of CN in transdermal delivery is to address the phenomenon of Localized Transport Regions (LTRs). Many sonophoresis studies have demonstrated a few, seemingly random, discrete patches of skin (LTRs) experiencing far more permeabilization, and therefore receiving far more drug, than the rest of the sonicated area [182,187,188,189]. LTRs are thought to occur at locations of greater cavitation activity [187,188], and increasing LTR coverage of the skin is an important topic in the interest of maximizing delivered dose. Pre-seeding the skin’s surface with CN could allow much more uniform, repeatable cavitation activity and thereby LTR formation.

### 4.3. Wound Healing 

Oxygen is essential for wound healing in tissues. More than just a nutrient, it is required for oxidative metabolism and the regulation of many signal transduction pathways and immune cell activity [190]. Hypoxia can lead to chronic ischemic wounds [191]. Wound oxygenation is thus a key determinant of healing outcomes and often used as a metric for treatment plans, including whether amputation is required [192]. CN have the potential to be a very useful vehicle in delivering oxygen to wound sites.

Hyperbaric oxygen therapy (HBOT) and topical oxygen therapy (TOT) are the most common clinical treatments for mitigating hypoxia in wounds. HBOT, in which a patient breathes pure oxygen at pressures greater than atmospheric pressure to induce hyperoxygenation, carries a risk of oxygen organ toxicity due to oxidative stress and genotoxicity because it is not targeted [193]. TOT, in contrast, applies oxygen gas at 100% saturation directly to the wound bed, and is possible with lightweight wearable systems that utilize compression-stacked electrochemical oxygen generators [194]. While it has been demonstrated to increase oxygen partial pressure levels at the wound base centre and decrease wound size and healing time compared to patients who did not undergo the therapy [193], diffusion of oxygen is limited. Oxygen gas on its own can penetrate through the epidermis into the dermis, but at deeper dermis layers there is no change in oxygen concentration [195]. 

Perfluorocarbon (PFC) emulsions developed to absorb large amounts of oxygen have recently emerged as an alternative treatment option. When applied to a hypoxic area, the oxygen will diffuse down the concentration gradient, providing oxygen to the needed area. These emulsions have been found to reduce complications after skin procedures, decrease tissue hypoxia in phlegmons, increase the speed of local wound healing, and enhance the rate of epithelialization [196,197,198]. 

Researchers hypothesize that techniques used to deliver oxygen via CN could also improve wound healing applications. In 2015, Eisenbrey proposed that his technique of exposing oxygen-loaded microbubbles to ultrasound to increase oxygen content in breast tumors could benefit wound healing in the future [199]. In 2023, Ho et al. found that cavitation of oxygen-loaded microbubbles increased vasodilation and angiogenesis at ischaemia–perfusion vessels. Their proposed pathway of cavitation induced endothelial nitric oxide synthase (eNOS) activation, vascular endothelial growth factor (VEGF) expression, and a reduction in interstitial hydrogen peroxide, all instrumental in remodelling of the vascular architecture in the wound healing process. They thus hypothesized that this pathway could drive further investigation into wound healing and avoid the side effects induced by hyperbaric oxygen therapy [200]. 

Though cavitation-mediated oxygen delivery in wound healing has not yet been clinically tested, current applications in hydrogels and other biomaterial scaffolds show promise. Nanodroplets engineered to carry oxygen via haemoglobin encapsulation increased cell viability of cardiomyocytes in a GelMA hydrogel and expedited the repair of infarcted tissues when exposed to low-intensity pulsed ultrasound [201]. Preliminary work exploring the effect of oxygen-loaded microbubbles exposed to ultrasound on human dermal fibroblasts (HDFs) also found that they increased HDF viability [202]. As HDFs are one of the main cell types involved in the wound healing process, increased viability could aid in a timely repair process. 

PFCs have also been commonly used in CN [199,203,204,205,206]. PFC oxygen-loaded cavitation nuclei can be used to deliver oxygen to hypoxic regions of wound beds, combining the advantages of high-oxygen PFC emulsions with the added benefits of ultrasound-mediated cavitation to include temporal release, deeper penetration, and specific localization within the wound bed. Some physicians argue that oxygen therapy for wounds should be a multi-faceted approach in which oxygen is specifically dosed as a function of tissue hypoxia [191] and focused at the centre of the wound where hypoxia is typically greatest. Sonosensitive cavitation nuclei as a vehicle for oxygen delivery present a plausible solution to these challenges.

### 4.4. Biofilms

Between 65% and 80% of clinical bacterial infections involve biofilms: communities of microbes existing within a matrix of extracellular polymeric substances including proteins, polysaccharides, and extracellular DNA [207]. Biofilms present a major challenge to effective antibiotic treatments; not only do they form a physical barrier to antibiotic drug penetration [208], but they also promote functional changes associated with resistance, including slower growth rates and communal stress responses [209]. Because of this, bacteria residing in biofilms are known to be 10–1000 times more resistant to antibiotics than planktonic bacteria [210]. As emerging pathogens become increasingly resistant to new antibiotics, novel strategies of treating infections by targeting the biofilm matrix have gained momentum, such as through biofilm-degrading enzymes [211] or, more recently, ultrasound in combination with CN [212].

CN have been shown to be effective in treating a wide variety of infections, including in vitro monospecies biofilms of both Gram-positive [213,214] and Gram-negative [215,216] bacterial species as well as more complex models, such as an infected clot model [217], a bladder organoid model [218], and an in vivo infected catheter model [219]. In all cases, the benefit of applying CN to a biofilm is twofold; first, cavitation can disrupt the structural integrity of the biofilm matrix via production of craters [220] and micropores [221], and second, it can enhance the delivery of antibiotic drugs, including gentamicin [216,218,220], vancomycin [213,221], oxacillin [212], and streptomycin [220]. Most studies have found that the synergistic application of cavitation with an antibiotic is more effective than either cavitation or antibiotic administration alone, in part because dispersion changes the physiological state of bacteria [222] and most antibiotic drugs are effective only on cells that are metabolically active [223,224]. That being said, dispersed bacteria may also be more virulent and more likely to reinfect than their planktonic counterparts [222]. 

The ease of functionalization of CN has also been explored in biofilm applications, primarily the conjugation of drugs and/or targeting ligands to lipid-shelled microbubbles. Vancomycin, in particular, has been explored both for its binding affinity and its cytotoxicity to Gram-positive bacteria [214], as its mode of action involves binding to the D-ala-D-ala moiety on the bacterial cell wall, hindering cell wall synthesis [225]. This theranostic strategy could theoretically be employed with other antibiotics that work by binding to surface receptors. Liposomes containing gentamicin have also been covalently attached to microbubbles, which has shown to result in higher intracellular delivery after ultrasound exposure than liposomal formulations of the drug alone [218]. Finally, “bioactive” gases have been evaluated for their ability to enhance the therapeutic potential of cavitation-mediated biofilm disruption and antibiotic delivery. Nitric oxide (NO) is a gaseous signalling molecule that has been shown to disperse biofilms at low concentrations and kill pathogens at high concentrations [226], but is limited by its short half-life and limited penetration [227]. Encapsulating NO into lipid-shelled microbubbles is therefore able to increase the bioavailability of NO and has shown to result in biofilm removal and clinically relevant log reductions in culturable bacteria when combined with ultrasound and gentamicin [215]. Although lingering questions remain regarding the downstream effects and overall safety of cavitation-mediated biofilm dispersion, the urgency of antibiotic resistance warrants continued research in this area.

### 4.5. Blood–Brain Barrier

The blood–brain barrier is a key feature of the vasculature of the brain and consists of a layer of endothelial cells sealed together by specialized cell–cell junctions and supported by other cell types, including pericytes and astrocytes. The cells comprising the BBB have an exceptionally low number of transcytosis vesicles, specialized drug metabolizing enzymes, and efflux pumps to expel potentially toxic substances from brain to blood, culminating in a dynamic physical and metabolic barrier [228,229,230,231]. In tandem, specific transporters and carrier proteins are enriched in brain endothelial cells to facilitate controlled transport of specific essential nutrients and metabolites, such as glucose (GLUT-1), amino acids (LAD1), and transferrin [232]. The tight control of transport in both directions prevents the entry of most substances from the systemic blood supply, including leukocytes, into the brain, which helps maintain the physiological conditions required for neural signalling, and to shield neural tissues from neurotoxins in the blood [233]. Unfortunately, the BBB also acts as the major bottleneck in drug delivery to treat diseases in the central nervous system (CNS) [233]; 98% of small-molecule drugs, and all biologics, cannot pass through the BBB into a non-disrupted brain unaided [234]. This presents a significant barrier to delivering a meaningful concentration of drugs to treat neurological diseases, which were the second leading cause of death between 1990 and 2016, including psychiatric diseases, neurodegenerative diseases, brain cancers, and strokes. 

The combination of ultrasound and multiple types of CN has been shown to non-invasively and reversibly open the BBB to achieve the delivery of macromolecular therapeutic drugs such as monoclonal antibodies, genes, and chemotherapies into the brain parenchyma [235,236,237]. The ability to focus the US beam down to the millimetre scale provides much more spatiotemporal precision and control than alternative approaches, such as co-administration of vasodilators or hyperosmotic agents, Trojan horse (MAb conjugation), etc., and hence limits the risk of off-target adverse effects in the brain [238]. US + CN can be combined with real-time stereotactic image guidance or acoustic emission monitoring to provide feedback on efficacy and risk, allowing fine tuning of the US dose during treatment [238,239].

### 4.6. Gastrointestinal Drug Delivery 

Due to its convenience, non-invasiveness, and ease of use, oral drug delivery is the preferred route of administration for patients [240]. The two major barriers to effective oral delivery of biopharmaceuticals includes their instability in the gastrointestinal tract, and their erratic absorption across the intestinal membrane into systemic circulation. Permeability is limited by two physiological barriers: the mucus layer and the epithelial layer. The mucus layer slows the diffusion rate of large molecules, whilst the epithelium prevents the diffusion of large molecules [241,242]. 

It is hypothesized that the application of ultrasound to the gastrointestinal tract could provide a means of rapidly delivering small molecules, but also facilitate the delivery of macromolecules across the mucus and epithelial layers of the gastrointestinal tract [243]. Ultrasound-mediated gastrointestinal drug delivery research is largely influenced by the transdermal drug delivery literature, which has highlighted the potential of low-frequency ultrasound to facilitate drug delivery [244,245,246,247,248,249,250,251].

Schoellhammer et al. demonstrated the safety and tolerance of low-frequency ultrasound-mediated drug delivery of small and large molecules in vitro and in vivo, showing successful delivery in both the rectum and buccal cavity without cavitation nuclei [252,253,254,255,256]. Stewart et al. reported on the development of a multimodal diagnostic endoscopic capsule device, with high-frequency quantitative micro-ultrasound complementing video imaging, allowing subsurface visualization and computer-assisted diagnosis. The studies showed that the application of ultrasound to Caco-2 cell monolayers alone and ultrasound combined with Sonovue microbubbles (Sonovue^®^, phospholipid coating with sulfur hexafluoride gas core; Bracco diagnostics, Inc., Milan, Italy) decreased transepithelial electrical resistance, suggesting permeabilization of the cell layer. The team also demonstrated the ability to direct microbubble streams to the focus of the transducer using acoustic radiation forces [257,258]. 

Using a modified version of the prototype capsule reported previously, ultrasound-mediated targeted drug delivery of quantum dots has been demonstrated with ex vivo tissue and in vivo. Fluorescent markers chosen as a model drug were used to demonstrate in vivo delivery, using a porcine small intestine with this capsule in vivo. The fluorescent markers combined with microbubbles and focused ultrasound were shown to penetrate the mucus layer of the small intestine. These findings illustrate the potential of this device for ultrasound-mediated gastrointestinal drug delivery, and the challenges (e.g., tethering of capsule, debris lodged into outlets) to be overcome before focused ultrasound and microbubbles could be used with this device for the oral delivery of biopharmaceuticals [259]. 

Fix et al. evaluated the potential of using ultrasound-stimulated phase-change ultrasound contrast agents (1,2-distearoylsn-glycero-3-phosphocholine and 1,2-distearoyl-snglycero-3-phosphoethanolamine-N-methoxy(polyethyleneglycol)-2000 with octofluoropropane gas) to cause transient disruption of Caco-2 epithelial monolayers cultured on permeable Transwell supports and enhance the permeation of a model macromolecular drug. The team assessed ultrasound-mediated drug delivery through Caco-2 monolayers where ultrasound and phase-change ultrasound contrast agents combined were found to enhance dextran delivery in comparison to the negligible amount delivered in the control samples (phase-change ultrasound contrast agents alone; ultrasound alone) [260]. 

In summary, ultrasound-mediated gastrointestinal drug delivery is a nascent field with many unexplored research questions, but the flexibility of the various CN described above could be of enormous benefit. 

## 5. Future Directions of the Field

Cavitation-enhanced drug delivery has extraordinary potential as a tool to overcome a wide range of physiological and biological barriers, including solid tumors, the skin, the gastro-intestinal mucosa, the blood–brain barrier, and biofilms. Its application is relatively simple and can be non-invasive through the use of extracorporeal or interstitial ultrasound transducers, involving no ionizing radiation and no significant side effects other than those potentially associated with the thermal and mechanical effects of ultrasound. The use of exogenous cavitation nuclei, be they solid CN, microbubbles, nanodroplets, echogenic liposomes, or vesicles, also makes it possible to enhance the delivery, penetration, and distribution of therapeutics without making it necessary to modify them in any way. Conversely, CN can also be readily modified to incorporate or present the therapeutic as demanded by specific applications for optimal interaction with the cell or therapeutic target.

Until recently, this exciting new approach was primarily confined to pre-clinical efforts, but numerous recent advances have seen the initiation and successful completion of several clinical trials evidencing significant improvements in therapeutic efficacy, reductions in systemic and off-target toxicity, and the potential opportunity to reduce the overall dose of drugs administered to the patient whilst still achieving equivalent or greater therapeutic benefit. These clinical trials are also actively gathering essential information in terms of cavitation monitoring and mapping, which are enabling the establishment of clinically relevant safety and efficacy metrics that can now be monitored in real time during drug administration. If successful, these metrics could provide a means of confirming for the very first time that the CN, and therefore by extension the co-administered therapeutic, has successfully reached its target.

In spite of these great strides, several challenges and opportunities still remain. The majority of CN developed to date are primarily optimized to support acoustic cavitation, but there is a growing trend towards designing gas carriers which also enhance or facilitate the very therapeutic process they are intended to support, in addition to generating the mechanical and fluidic forces associated with cavitation. CN optimization greatly depends on the development of a deeper scientific understanding of the interaction between cavitating bubbles and adjacent biological structures, notably in terms of the differentiated biological, chemical, inflammatory, and immunological processes known to take place in the presence of elevated shear stresses and oscillatory forces. Widespread clinical adoption is reliant on improved and robust scalable manufacturing techniques, that not only enable production with minimal batch-to-batch variability but also facile yet reliable modification of formulations to target different indications or drugs as needed. Furthermore, widely accepted safety and efficacy ranges using standardized metrics based on real-time cavitation monitoring/mapping need to be established by indication, on the basis of the early clinical data presently being gathered. Lastly, several regulatory and commercial challenges remain, in particular on whether ultrasound-enhanced drug delivery strategies are seen merely as a device supplementing the mode of action of a drug, or as a more complex combination which introduces novel and potentially exciting new modes of action. The fields of ultrasound-enhanced drug delivery, immunomodulation, immune-oncology, transdermal vaccination, and antimicrobial therapies are as young as they are promising, and their success will ultimately rely on our ability to address these key challenges and identify clinical indications where these approaches have a transformative impact for the benefit of patients. 

## Figures and Tables

**Table 1:** Overview of clinical trials involving ultrasound-enhanced drug delivery to extra-cranial solid tumors.

Tumor Site	Study Design	n	Cavitation Agent	Anti-Cancer Agent	US Modality	ClinicalTrials.Gov or ISCRTN ID
**Colorectal liver metastases**	Phase 1–2	45	SonoTran^®^ Particles (OxsSonics) (nanocups)	FOLFIRI and Cetuximab	Focused ultrasound	ISRCTN17598292 [170]
**Solid tumours with liver metastases (colon and pancreas)**	Phase 1–2	37–43	Microbubble–microdroplet clusters (PS101)	FOLFOX/FOLFIRI for colorectal cancer; Gemcitabine and nab-paclitaxel for pancreatic cancer	Dual-frequency ultrasound	NCT04021277 [171]
**Breast**	Window-of-opportunity study, randomized	48	No cavitation agent	Gemcitabine	Focused ultrasound	NCT04796220 [172]
**Liver metastases from breast and colon/rectum**	2 liver metastases in each patient; 1 target lesion and the other control lesion	60	SonoVue^®^ (Bracco) Microbubbles	Chemotherapy not otherwise specified	Focused ultrasound	NCT03477019 [173]
**Metastatic colorectal cancer**	Early phase, single arm	10	No cavitation agent	Toripalimab and regorafenib	HIFU	NCT04819516 [174]
**Locally advanced pancreatic cancer**	Early-phase, single-arm, exploratory clinical trial	60	No cavitation agent	FOLFIRINOX	HIFU	NCT05262452 [175]
**Pancreas**	Phase I/II, randomized	120	Sonazoid microbubbles	FOLFIRINOX	Focused ultrasound	NCT04821284 [176]
**Inoperable pancreatic ductal adenocarcinoma**	Early phase, randomized	30	SonoVue Microbubbles	Chemotherapy, not otherwise specified	Focused ultrasound	NCT04146441 [177]
**Pediatric refractory solid tumor**	Phase 1, non-randomized	34	No cavitation agent	ThermoDox	MR-HIFU	NCT02536183 [178]

## References

[1] Scannell J.W., Blanckley A., Boldon H., Warrington B. (2012). Diagnosing the decline in pharmaceutical R&D efficiency. Nat. Rev. Drug Discov..

[2] May M. (2022). Why drug delivery is the key to new medicines. Nat. Med..

[3] Surapaneni M.S., Das S.K., Das N.G. (2012). Designing Paclitaxel drug delivery systems aimed at improved patient outcomes: Current status and challenges. Int. Sch. Res. Not..

[4] Mitchell M.J., Billingsley M.M., Haley R.M., Wechsler M.E., Peppas N.A., Langer R. (2021). Engineering precision nanoparticles for drug delivery. Nat. Rev. Drug Discov..

[5] Prabhakar U., Maeda H., Jain R.K., Sevick-Muraca E.M., Zamboni W., Farokhzad O.C., Barry S.T., Gabizon A., Grodzinski P. (2013). Challenges and key considerations of the enhanced permeability and retention effect for nanomedicine drug deliery in oncology. Amer. Assoc. Canc. Res..

[6] Manzoor A.A., Lindner L.H., Landon C.D., Park J.Y., Simnick A.J., Dreher M.R., Das S., Hanna G., Park W., Chilkoti A. (2012). Overcoming limitations in nanoparticle drug delivery: Triggered, intravascular release to improve drug penetration into tumors. Cancer Res..

[7] Correia J.H., Rodrigues J.A., Pimenta S., Dong T., Yang Z. (2021). Photodynamic Therapy Review: Principles, Photosensitizers, Applications, and Future Directions. Pharmaceutics.

[8] Dou Y., Hynynen K., Allen C. (2017). To heat or not to heat: Challenges with clinical translation of thermosensitive liposomes. J. Control. Release.

[9] Garello F., Svenskaya Y., Parakhonskiy B., Filippi M. (2023). On the Road to Precision Medicine: Magnetic Systems for Tissue Regeneration, Drug Delivery, Imaging, and Theranostics. Pharmaceutics.

[10] Coussios C.C., Roy R.A. (2008). Applications of Acoustics and Cavitation to Noninvasive Therapy and Drug Delivery. Annu. Rev. Fluid Mech..

[11] Stride E., Coussios C. (2019). Nucleation, mapping and control of cavitation for drug delivery. Nat. Rev. Phys..

[12] Elder S.A. (1959). Cavitation microstreaming. J. Acoust. Soc. Am..

[13] Tho P., Manasseh R., Ooi A. (2007). Cavitation microstreaming patterns in single and multiple bubble systems. J. Fluid Mech..

[14] Tzu-Yin W., Wilson K.E., Machtaler S., Willmann J.K. (2013). Ultrasound and microbubble guided drug delivery: Mechanistic understanding and clinical implications. Curr. Pharm. Biotechnol..

[15] Hu Y., Wan J.M.F., Yu A.C.H. (2013). Membrane Perforation and Recovery Dynamics in Microbubble-Mediated Sonoporation. Ultrasound Med. Biol..

[16] Flannigan D.J., Suslick K.S. (2005). Plasma formation and temperature measurement during single-bubble cavitation. Nature.

[17] Gaitan D.F., Crum L.A., Church C.C., Roy R.A. (1992). Sonoluminescence and bubble dynamics for a single, stable, cavitation bubble. J. Acoust. Soc. Am..

[18] Marinesco N. (1933). Action des ultrasons sur les plaques photographiques. Proc. R. Acad. Sci. Amst..

[19] Hilgenfeldt S., Grossmann S., Lohse D. (1999). A simple explanation of light emission in sonoluminescence. Nature.

[20] Pecha R., Gompf B. (2000). Microimplosions: Cavitation Collapse and Shock Wave Emission on a Nanosecond Time Scale. Phys. Rev. Lett..

[21] Holzfuss J., Rüggeberg M., Billo A. (1998). Shock Wave Emissions of a Sonoluminescing Bubble. Phys. Rev. Lett..

[22] Suslick K.S., Doktycz S.J., Flint E.B. (1990). On the origin of sonoluminescence and sonochemistry. Ultrasonics.

[23] Makino K., Mossoba M.M., Riesz P. (1982). Chemical effects of ultrasound on aqueous solutions. Evidence for hydroxyl and hydrogen free radicals (.cntdot.OH and .cntdot.H) by spin trapping. J. Am. Chem. Soc..

[24] Maardalen M., Carlisle R., Coussios C. (2023). Cavitation-Mediated Immunomodulation and Its Use with Checkpoint Inhibitors. Pharmaceutics.

[25] Edsall C., Ham E., Holmes H., Hall T.L., Vlaisavljevich E. (2021). Effects of frequency on bubble-cloud behavior and ablation efficiency in intrinsic threshold histotripsy. Phys. Med. Biol..

[26] Apfel R.E. (1984). Acoustic cavitation inception. Ultrasonics.

[27] Karamanou M., Papaioannou T.G., Stefanadis C., Androutsos G. (2012). Genesis of ultrasonic microbubbles: A quick historical overview. Curr. Pharm. Des..

[28] Kooiman K., Vos H.J., Versluis M., de Jong N. (2014). Acoustic behavior of microbubbles and implications for drug delivery. Adv. Drug Deliv. Rev..

[29] Roovers S., Segers T., Lajoinie G., Deprez J., Versluis M., De Smedt S.C., Lentacker I. (2019). The Role of Ultrasound-Driven Microbubble Dynamics in Drug Delivery: From Microbubble Fundamentals to Clinical Translation. Langmuir.

[30] Yusefi H., Helfield B. (2022). Ultrasound Contrast Imaging: Fundamentals and Emerging Technology. Front. Phys..

[31] Sirsi S., Borden M. (2009). Microbubble Compositions, Properties and Biomedical Applications. Bubble Sci. Eng. Technol..

[32] Ibsen S., Schutt C.E., Esener S. (2013). Microbubble-mediated ultrasound therapy: A review of its potential in cancer treatment. Drug Des. Dev. Ther..

[33] McEwan C., Owen J., Stride E., Fowley C., Nesbitt H., Cochrane D., Coussios C.C., Borden M., Nomikou N., McHale A.P. (2015). Oxygen carrying microbubbles for enhanced sonodynamic therapy of hypoxic tumours. J. Control. Release.

[34] Lee H., Kim H., Han H., Lee M., Lee S., Yoo H., Chang J.H., Kim H. (2017). Microbubbles used for contrast enhanced ultrasound and theragnosis: A review of principles to applications. Biomed. Eng. Lett..

[35] Kwan J.J., Borden M.A. (2012). Lipid monolayer collapse and microbubble stability. Adv. Colloid Interface Sci..

[36] Borden M.A. (2019). Intermolecular Forces Model for Lipid Microbubble Shells. Langmuir.

[37] Pellow C., Abenojar E.C., Exner A.A., Zheng G., Goertz D.E. (2020). Concurrent visual and acoustic tracking of passive and active delivery of nanobubbles to tumors. Theranostics.

[38] Xing Z., Wang J., Ke H., Zhao B., Yue X., Dai Z., Liu J. (2010). The fabrication of novel nanobubble ultrasound contrast agent for potential tumor imaging. Nanotechnology.

[39] Myers J.Z., Navarro-Becerra J.A., Borden M.A. (2022). Nanobubbles are non-echogenic for fundamental-mode contrast-enhanced ultrasound imaging. Bioconjugate Chem..

[40] Upadhyay A., Dalvi S.V., Gupta G., Khanna N. (2017). Effect of PEGylation on performance of protein microbubbles and its comparison with lipid microbubbles. Mater. Sci. Eng. C.

[41] Rudakovskaya P.G., Barmin R.A., Kuzmin P.S., Fedotkina E.P., Sencha A.N., Gorin D.A. (2022). Microbubbles Stabilized by Protein Shell: From Pioneering Ultrasound Contrast Agents to Advanced Theranostic Systems. Pharmaceutics.

[42] Grinstaff M.W., Suslick K.S. (1991). Air-filled proteinaceous microbubbles: Synthesis of an echo-contrast agent. Proc. Natl. Acad. Sci. USA.

[43] Koczera P., Appold L., Shi Y., Liu M., Dasgupta A., Pathak V., Ojha T., Fokong S., Wu Z., van Zandvoort M. (2017). PBCA-based polymeric microbubbles for molecular imaging and drug delivery. J. Control. Release.

[44] Xiong X., Zhao F., Shi M., Yang H., Liu Y. (2011). Polymeric Microbubbles for Ultrasonic Molecular Imaging and Targeted Therapeutics. J. Biomater. Sci. Polym. Ed..

[45] Bloch S.H., Wan M., Dayton P.A., Ferrara K.W. (2004). Optical observation of lipid- and polymer-shelled ultrasound microbubble contrast agents. Appl. Phys. Lett..

[46] Xu Q., Nakajima M., Ichikawa S., Nakamura N., Shiina T. (2008). A comparative study of microbubble generation by mechanical agitation and sonication. Innov. Food Sci. Emerg. Technol..

[47] Bjerknes K., Sontum P.C., Smistad G., Agerkvist I. (1997). Preparation of polymeric microbubbles: Formulation studies and product characterisation. Int. J. Pharm..

[48] Suslick K.S., Didenko Y., Fang M.M., Hyeon T., Kolbeck K.J., McNamara W.B., Mdleleni M.M., Wong M. (1999). Acoustic cavitation and its chemical consequences. Philos. Trans. R. Soc. Lond. Ser. A Math. Phys. Eng. Sci..

[49] Thomson L.M., Polizzotti B.D., McGowan F.X., Kheir J.N. (2014). Manufacture of concentrated, lipid-based oxygen microbubble emulsions by high shear homogenization and serial concentration. J. Vis. Exp..

[50] Stride E., Edirisinghe M. (2008). Novel microbubble preparation technologies. Soft Matter.

[51] Pancholi K.P., Farook U., Moaleji R., Stride E., Edirisinghe M.J. (2008). Novel methods for preparing phospholipid coated microbubbles. Eur. Biophys. J..

[52] Joscelyne S.M., Trägårdh G. (2000). Membrane emulsification—A literature review. J. Membr. Sci..

[53] Jerri H.A., Dutter R.A., Velegol D. (2009). Fabrication of stable anisotropic microcapsules. Soft Matter.

[54] Farook U., Zhang H.B., Edirisinghe M.J., Stride E., Saffari N. (2007). Preparation of microbubble suspensions by co-axial electrohydrodynamic atomization. Med. Eng. Phys..

[55] Xia Y., Pack D.W. (2015). Uniform biodegradable microparticle systems for controlled release. Chem. Eng. Sci..

[56] Lee M., Lee E.Y., Lee D., Park B.J. (2015). Stabilization and fabrication of microbubbles: Applications for medical purposes and functional materials. Soft Matter.

[57] Zhang W., Shi Y., Abd Shukor S., Vijayakumaran A., Vlatakis S., Wright M., Thanou M. (2022). Phase-shift nanodroplets as an emerging sonoresponsive nanomaterial for imaging and drug delivery applications. Nanoscale.

[58] Loskutova K., Grishenkov D., Ghorbani M. (2019). Review on Acoustic Droplet Vaporization in Ultrasound Diagnostics and Therapeutics. BioMed. Res. Int..

[59] Choudhury S.A.B.S., Xie F.M.D., Dayton P.A.P., Porter T.R.M.D. (2017). Acoustic Behavior of a Reactivated, Commercially Available Ultrasound Contrast Agent. J. Am. Soc. Echocardiogr..

[60] Reznik N., Lajoinie G., Shpak O., Gelderblom E.C., Williams R., de Jong N., Versluis M., Burns P.N. (2014). On the Acoustic Properties of Vaporized Submicron Perfluorocarbon Droplets. Ultrasound Med. Biol..

[61] Keipert P.E., Otto S., Flaim S.F., Weers J.G., Schutt E.A., Pelura T.J., Klein D.H., Yaksh T.L. (1994). Influence of Perflubron Emulsion Particle Size on Blood Half-Life and Febrile Response in Rats. Artif. Cells Blood Substit. Biotechnol..

[62] Navarro-Becerra J.A., Song K.H., Martinez P., Borden M.A. (2022). Microbubble Size and Dose Effects on Pharmacokinetics. ACS Biomater. Sci. Eng..

[63] Borden M.A., Shakya G., Upadhyay A., Song K.-H. (2020). Acoustic nanodrops for biomedical applications. Curr. Opin. Colloid Interface Sci..

[64] Vlaisavljevich E., Aydin O., Durmaz Y.Y., Lin K.W., Fowlkes B., Xu Z., ElSayed M.E. (2016). Effects of Droplet Composition on Nanodroplet-Mediated Histotripsy. Ultrasound Med. Biol..

[65] Mountford P.A., Borden M.A. (2016). On the thermodynamics and kinetics of superheated fluorocarbon phase-change agents. Adv. Colloid Interface Sci..

[66] Lee J.Y., Carugo D., Crake C., Owen J., de Saint Victor M., Seth A., Coussios C., Stride E. (2015). Nanoparticle-Loaded Protein–Polymer Nanodroplets for Improved Stability and Conversion Efficiency in Ultrasound Imaging and Drug Delivery. Adv. Mater..

[67] Bau L., Wu Q., Smith C.A.B., Reimer K., Tang M., Ovenden N., Stride E. Predicting the spontaneous vaporisation of nanodroplets. Proceedings of the 28th European Symposium on Ultrasound Contrast Imaging.

[68] Kim J., DeRuiter R.M., Goel L., Xu Z., Jiang X., Dayton P.A. (2020). A Comparison of Sonothrombolysis in Aged Clots between Low-Boiling-Point Phase-Change Nanodroplets and Microbubbles of the Same Composition. Ultrasound Med. Biol..

[69] Mannaris C., Bau L., Grundy M., Gray M., Lea-Banks H., Seth A., Teo B., Carlisle R., Stride E., Coussios C.C. (2019). Microbubbles, nanodroplets and gas-stabilizing solid particles for ultrasound-mediated extravasation of unencapsulated drugs: An exposure parameter optimization study. Ultrasound Med. Biol..

[70] Mountford P.A., Sirsi S.R., Borden M.A. (2014). Condensation phase diagrams for lipid-coated perfluorobutane microbubbles. Langmuir.

[71] Sheeran P.S., Matsunaga T.O., Dayton P.A. (2013). Phase-transition thresholds and vaporization phenomena for ultrasound phase-change nanoemulsions assessed via high-speed optical microscopy. Phys. Med. Biol..

[72] Welch P.J., Li D.S., Forest C.R., Pozzo L.D., Shi C. (2022). Perfluorocarbon nanodroplet size, acoustic vaporization, and inertial cavitation affected by lipid shell composition in vitro. J. Acoust. Soc. Am..

[73] Fabiilli M.L., Haworth K.J., Fakhri N.H., Kripfgans O.D., Carson P.L., Fowlkes J.B. (2009). The role of inertial cavitation in acoustic droplet vaporization. IEEE Trans. Ultrason. Ferroelectr. Freq. Control.

[74] Kripfgans O.D., Fowlkes J.B., Miller D.L., Eldevik O.P., Carson P.L. (2000). Acoustic droplet vaporization for therapeutic and diagnostic applications. Ultrasound. Med. Biol..

[75] Vasiukhina A., Eshraghi J., Ahmadzadegan A., Goergen C.J., Vlachos P.P., Solorio L. (2021). Stable Thermally-Modulated Nanodroplet Ultrasound Contrast Agents. Nanomaterials.

[76] Huang Y., Vezeridis A.M., Wang J., Wang Z., Thompson M., Mattrey R.F., Gianneschi N.C. (2017). Polymer-Stabilized Perfluorobutane Nanodroplets for Ultrasound Imaging Agents. J. Am. Chem. Soc..

[77] Yuksel Durmaz Y., Vlaisavljevich E., Xu Z., ElSayed M. (2014). Development of Nanodroplets for Histotripsy-Mediated Cell Ablation. Mol. Pharm..

[78] Baghbani F., Chegeni M., Moztarzadeh F., Hadian-Ghazvini S., Raz M. (2017). Novel ultrasound-responsive chitosan/perfluorohexane nanodroplets for image-guided smart delivery of an anticancer agent: Curcumin. Mater. Sci. Eng. C.

[79] Lea-Banks H., O’Reilly M.A., Hynynen K. (2019). Ultrasound-responsive droplets for therapy: A review. J. Control. Release.

[80] Rapoport N.Y., Kennedy A.M., Shea J.E., Scaife C.L., Nam K.-H. (2009). Controlled and targeted tumor chemotherapy by ultrasound-activated nanoemulsions/microbubbles. J. Control. Release.

[81] Li D.S., Schneewind S., Bruce M., Khaing Z., O’Donnell M., Pozzo L. (2019). Spontaneous Nucleation of Stable Perfluorocarbon Emulsions for Ultrasound Contrast Agents. Nano Lett..

[82] Sheeran P.S., Rojas J.D., Puett C., Hjelmquist J., Arena C.B., Dayton P.A. (2015). Contrast-Enhanced Ultrasound Imaging and in Vivo Circulatory Kinetics with Low-Boiling-Point Nanoscale Phase-Change Perfluorocarbon Agents. Ultrasound Med. Biol..

[83] Wallqvist V., Claesson P.M., Swerin A., Schoelkopf J., Gane P.A. (2006). Interaction forces between talc and hydrophobic particles probed by AFM. Colloids Surf. A Physicochem. Eng. Asp..

[84] Kwan J.J., Myers R., Coviello C.M., Graham S.M., Shah A.R., Stride E., Carlisle R.C., Coussios C.C. (2015). Ultrasound-propelled nanocups for drug delivery. Small.

[85] Su X., Thomas R.G., Bharatula L.D., Kwan J.J. (2019). Remote targeted implantation of sound-sensitive biodegradable multi-cavity microparticles with focused ultrasound. Sci. Rep..

[86] Hettinga J.K., Lyons B., Balkaran J., Rijal P., Dunn-Lawless D., Caproni L., Gray M., Suslick K.S., Coussios C.C., Carlisle R.C. (2023). Cavitation-mediated Transcutaneous Delivery of Protein and Nucleotide-based Antigen for Rapid High-level Immune Responses. Adv. Ther..

[87] Zhang P., He J., Ma X., Gong J., Nie Z. (2013). Ultrasound assisted interfacial synthesis of gold nanocones. Chem. Commun..

[88] Oxley J.D., Prozorov T., Suslick K.S. (2003). Sonochemistry and sonoluminescence of room-temperature ionic liquids. J. Am. Chem. Soc..

[89] Suslick K.S., Doctycz S. (1990). Interparticle collisions driven by ultrasound. Science.

[90] Xu H., Zeiger B.W., Suslick K.S. (2013). Sonochemical synthesis of nanomaterials. Chem. Soc. Rev..

[91] Bang J.H., Suslick K.S. (2010). Applications of ultrasound to the synthesis of nanostructured materials. Adv. Mater..

[92] Su X., Jonnalagadda U.S., Bharatula L.D., Kwan J.J. (2021). Unsupported gold nanocones as sonocatalytic agents with enhanced catalytic properties. Ultrason. Sonochem..

[93] Sazgarnia A., Shanei A., Taheri A.R., Meibodi N.T., Eshghi H., Attaran N., Shanei M.M. (2013). Therapeutic effects of acoustic cavitation in the presence of gold nanoparticles on a colon tumor model. J. Ultrasound Med..

[94] Kresge A.C., Leonowicz M.E., Roth W.J., Vartuli J., Beck J. (1992). Ordered mesoporous molecular sieves synthesized by a liquid-crystal template mechanism. Nature.

[95] Beck J.S., Vartuli J.C., Roth W.J., Leonowicz M.E., Kresge C.T., Schmitt K.D., Chu C.T.W., Olson D.H., Sheppard E.W., McCullen S.B. (1992). A new family of mesoporous molecular sieves prepared with liquid crystal templates. J. Am. Chem. Soc..

[96] Manzano M., Vallet-Regí M. (2019). Ultrasound responsive mesoporous silica nanoparticles for biomedical applications. Chem. Commun..

[97] Hah H.J., Kim J.S., Jeon B.J., Koo S.M., Lee Y.E. (2003). Simple preparation of monodisperse hollow silica particles without using templates. Chem. Commun..

[98] Paris J.L., Mannaris C., Cabañas M.V., Carlisle R., Manzano M., Vallet-Regí M., Coussios C.C. (2018). Ultrasound-mediated cavitation-enhanced extravasation of mesoporous silica nanoparticles for controlled-release drug delivery. Chem. Eng. J..

[99] Sviridov A., Tamarov K., Fesenko I., Xu W., Andreev V., Timoshenko V., Lehto V.P. (2019). Cavitation induced by Janus-like mesoporous silicon nanoparticles enhances ultrasound hyperthermia. Front. Chem..

[100] Kim H.J., Matsuda H., Zhou H., Honma I. (2006). Ultrasound-triggered smart drug release from a poly(dimethylsiloxane)-mesoporous silica composite. Adv. Mater..

[101] Lee S.F., Zhu X.M., Wang Y.X.J., Xuan S.H., You Q., Chan W.H., Wong C.H., Wang F., Yu J.C., Cheng C.H.K. (2013). Ultrasound, pH, and magnetically responsive crown-ether-coated core/shell nanoparticles as drug encapsulation and release systems. ACS Appl. Mater. Interfaces.

[102] Milgroom A., Intrator M., Madhavan K., Mazzaro L., Shandas R., Liu B., Park D. (2014). Mesoporous silica nanoparticles as a breast-cancer targeting ultrasound contrast agent. Colloids Surf. B Biointerfaces.

[103] Ma M., Xu H., Chen H., Jia X., Zhang K., Wang Q., Zheng S., Wu R., Yao M., Cai X. (2014). A drug–perfluorocarbon nanoemulsion with an ultrathin silica coating for the synergistic effect of chemotherapy and ablation by high-intensity focused ultrasound. Adv. Mater..

[104] Farjadian F., Roointan A., Mohammadi-Samani S., Hosseini M. (2019). Mesoporous silica nanoparticles: Synthesis, pharmaceutical applications, biodistribution, and biosafety assessment. Chem. Eng. J..

[105] Holland C.K., McPherson D.D. (2009). Echogenic Lipsomes for Targeted Drug Delivery. Proc. IEEE Int. Symp. Biomed. Imaging.

[106] Wei M., Lai M., Zhang J., Pei X., Yan F. (2022). Biosynthetic Gas Vesicles from Halobacteria NRC-1: A Potential Ultrasound Contrast Agent for Tumor Imaging. Pharmaceutics.

[107] Park D., Jung H.C., Park J., Bae S., Shin U., Kim S.W., Kim C.W., Lee Y.H., Seo J. (2022). Synthesis of echogenic liposomes for sonoporation. Micro Nano Lett..

[108] Bar-Zion A., Nourmahnad A., Mittelstein D.R., Shivaei S., Yoo S., Buss M.T., Hurt R.C., Malounda D., Abedi M.H., Lee-Gosselin A. (2021). Acoustically triggered mechanotherapy using genetically encoded gas vesicles. Nat. Nanotechnol..

[109] Dasgupta A., Sun T., Palomba R., Rama E., Zhang Y., Power C., Moeckel D., Liu M., Sarode A., Weiler M. (2023). Nonspherical ultrasound microbubbles. Proc. Natl. Acad. Sci. USA.

[110] Oh J.Y., Yang G., Choi E., Ryu J.-H. (2022). Mesoporous silica nanoparticle-supported nanocarriers with enhanced drug loading, encapsulation stability, and targeting efficiency. Biomater. Sci..

[111] Escoffre J.M., Piron J., Novell A., Bouakaz A. (2011). Doxorubicin Delivery into Tumor Cells with Ultrasound and Microbubbles. Mol. Pharm..

[112] Kotopoulis S., Dimcevski G., Helge Gilja O., Hoem D., Postema M. (2013). Treatment of human pancreatic cancer using combined ultrasound, microbubbles, and gemcitabine: A clinical case study. Med. Phys..

[113] Qiu Y., Luo Y., Zhang Y., Cui W., Zhang D., Wu J., Zhang J., Tu J. (2010). The correlation between acoustic cavitation and sonoporation involved in ultrasound-mediated DNA transfection with polyethylenimine (PEI) in vitro. J. Control. Release.

[114] Meijering B.D., Juffermans L.J., van Wamel A., Henning R.H., Zuhorn I.S., Emmer M., Versteilen A.M.G., Paulus W.J., van Gilst W.H., Kooiman K. (2009). Ultrasound and microbubble-targeted delivery of macromolecules is regulated by induction of endocytosis and pore formation. Circ. Res..

[115] Howard C.M., Forsberg F., Minimo C., Liu J.B., Merton D.A., Claudio P.P. (2006). Ultrasound guided site specific gene delivery system using adenoviral vectors and commercial ultrasound contrast agents. J. Cell. Physiol..

[116] Myers R., Coviello C., Erbs P., Foloppe J., Rowe C., Kwan J., Crake C., Finn S., Jackson E., Balloul J.M. (2016). Polymeric Cups for Cavitation-mediated Delivery of Oncolytic Vaccinia Virus. Mol. Ther..

[117] Carlisle R., Choi J., Bazan-Peregrino M., Laga R., Subr V., Kostka L., Ulbrich K., Coussios C.C., Seymour L.W. (2013). Enhanced tumor uptake and penetration of virotherapy using polymer stealthing and focused ultrasound. J. Natl. Cancer Inst..

[118] Jin L.F., Li F., Wang H.P., Wei F., Qin P., Du L.F. (2013). Ultrasound targeted microbubble destruction stimulates cellular endocytosis in facilitation of adeno-associated virus delivery. Int. J. Mol. Sci..

[119] Rychak J.J., Klibanov A.L. (2014). Nucleic acid delivery with microbubbles and ultrasound. Adv. Drug Deliv. Rev..

[120] Bao S., Thrall B.D., Miller D.L. (1997). Transfection of a reporter plasmid into cultured cells by sonoporation in vitro. Ultrasound Med. Biol..

[121] Lai C.-Y., Wu C.-H., Chen C.-C., Li P.-C. (2006). Quantitative relations of acoustic inertial cavitation with sonoporation and cell viability. Ultrasound Med. Biol..

[122] Dimcevski G., Kotopoulis S., Bjånes T., Hoem D., Schjøtt J., Gjertsen B.T., Biermann M., Molven A., Sorbye H., McCormack E. (2016). A human clinical trial using ultrasound and microbubbles to enhance gemcitabine treatment of inoperable pancreatic cancer. J. Control. Release Off. J. Control. Release Soc..

[123] Zhou B., Lian Q., Jin C., Lu J., Xu L., Gong X., Zhou P. (2022). Human clinical trial using diagnostic ultrasound and microbubbles to enhance neoadjuvant chemotherapy in HER2-negative breast cancer. Front. Oncol..

[124] Carpentier A., Canney M., Vignot A., Reina V., Beccaria K., Horodyckid C., Karachi C., Leclercq D., Lafon C., Chapelon J.Y. (2016). Clinical trial of blood-brain barrier disruption by pulsed ultrasound. Sci. Transl. Med..

[125] Lipsman N., Meng Y., Bethune A.J., Huang Y., Lam B., Masellis M., Herrmann N., Heyn C., Aubert I., Boutet A. (2018). Blood–brain barrier opening in Alzheimer’s disease using MR-guided focused ultrasound. Nat. Commun..

[126] Sletten E.M., Bertozzi C.R. (2011). From mechanism to mouse: A tale of two bioorthogonal reactions. Acc. Chem. Res..

[127] Stephanopoulos N., Francis M.B. (2011). Choosing an effective protein bioconjugation strategy. Nat. Chem. Biol..

[128] Benchimol M.J., Hsu M.J., Schutt C.E., Hall D.J., Mattrey R.F., Esener S.C. (2013). Phospholipid/carbocyanine dye-shelled microbubbles as ultrasound-modulated fluorescent contrast agents. Soft Matter.

[129] Borden M.A., Caskey C.F., Little E., Gillies R.J., Ferrara K.W. (2007). DNA and polylysine adsorption and multilayer construction onto cationic lipid-coated microbubbles. Langmuir.

[130] Dwivedi P., Kiran S., Han S., Dwivedi M., Khatik R., Fan R., Mangrio F.A., Du K., Zhu Z., Yang C. (2020). Magnetic Targeting and Ultrasound Activation of Liposome-Microbubble Conjugate for Enhanced Delivery of Anticancer Therapies. ACS Appl. Mater. Interfaces.

[131] Owen J., Rademeyer P., Chung D., Cheng Q., Holroyd D., Coussios C., Friend P., Pankhurst Q.A., Stride E. (2015). Magnetic targeting of microbubbles against physiologically relevant flow conditions. Interface Focus.

[132] Kolate A., Baradia D., Patil S., Vhora I., Kore G., Misra A. (2014). PEG—A versatile conjugating ligand for drugs and drug delivery systems. J. Control. Release.

[133] Wong M., Suslick K.S. (1994). Sonochemically produced hemoglobin microbubbles. MRS Online Proc. Libr..

[134] Cui Y., Dong H., Cai X., Wang D., Li Y. (2012). Mesoporous silica nanoparticles capped with disulfide-linked PEG gatekeepers for glutathione-mediated controlled release. ACS Appl. Mater. Interfaces.

[135] Zheng Z., Huang X., Schenderlein M., Borisova D., Cao R., Möhwald H., Shchukin D. (2013). Self-Healing and antifouling multifunctional coatings based on pH and sulfide ion sensitive nanocontainers. Adv. Funct. Mater..

[136] Mura S., Nicolas J., Couvreur P. (2013). Stimuli-responsive nanocarriers for drug delivery. Nat. Mater..

[137] Chapla R., Huynh K.T., Schutt C.E. (2022). Microbubble&ndash;Nanoparticle Complexes for Ultrasound-Enhanced Cargo Delivery. Pharmaceutics.

[138] Brown J.M., Wilson W.R. (2004). Exploiting tumour hypoxia in cancer treatment. Nat. Rev. Cancer.

[139] Fix S.M., Papadopoulou V., Velds H., Kasoji S.K., Rivera J.N., Borden M.A., Chang S., Dayton P.A. (2018). Oxygen microbubbles improve radiotherapy tumor control in a rat fibrosarcoma model–A preliminary study. PLoS ONE.

[140] Corro R., Urquijo C.F., Aguila O., Villa E., Santana J., Rios A., Escalante B. (2022). Use of nitric oxide donor-loaded microbubble destruction by ultrasound in thrombus treatment. Molecules.

[141] Abbott J.G. (1999). Rationale and derivation of MI and TI—A review. Ultrasound Med. Biol..

[142] Apfel R.E., Holland C.K. (1991). Gauging the likelihood of cavitation from short-pulse, low-duty cycle diagnostic ultrasound. Ultrasound Med. Biol..

[143] Bazan-Peregrino M., Rifai B., Carlisle R.C., Choi J., Arvanitis C.D., Seymour L.W., Coussios C.C. (2013). Cavitation-enhanced delivery of a replicating oncolytic adenovirus to tumors using focused ultrasound. J. Control. Release.

[144] DeImran K.M., Tintera B., Morrison H.A., Tupik J.D., Nagai-Singer M.A., Ivester H., Council-Troche M., Edwards M., Coutermarsh-Ott S., Byron C. (2023). Improved Therapeutic Delivery Targeting Clinically Relevant Orthotopic Human Pancreatic Tumors Engrafted in Immunocompromised Pigs Using Ultrasound-Induced Cavitation: A Pilot Study. Pharmaceutics.

[145] ISRCTN (2021). Testing If the Sonotran Platform Can Enhance Drug Delivery in Metastatic Colorectal Cancer. https://www.isrctn.com/ISRCTN17598292.

[146] Huang Y., Meng Y., Pople C.B., Bethune A., Jones R.M., Abrahao A., Hamani C., Kalia S.K., Kalia L.V., Lipsman N. (2022). Cavitation Feedback Control of Focused Ultrasound Blood-Brain Barrier Opening for Drug Delivery in Patients with Parkinson’s Disease. Pharmaceutics.

[147] Morse S.V., Pouliopoulos A.N., Chan T.G., Copping M.J., Lin J., Long N.J., Choi J.J. (2019). Rapid Short-pulse Ultrasound Delivers Drugs Uniformly across the Murine Blood-Brain Barrier with Negligible Disruption. Radiology.

[148] Edsall C., Khan Z.M., Mancia L., Hall S., Mustafa W., Johnsen E., Klibanov A.L., Durmaz Y.Y., Vlaisavljevich E. (2021). Bubble cloud behavoir and ablation capacity for histotripsy generated from intrinsic or artificial cavitation nuclei. Ultrasound Med. Biol..

[149] Li T., Khokhlova T.D., Sapozhnikov O.A., O’Donnell M., Hwang J.H. (2014). A New Active Cavitation Mapping Technique for Pulsed HIFU Applications-Bubble Doppler. IEEE Trans. Ultrason. Ferroelectr. Freq. Control.

[150] Gyöngy M., Coussios C.C. (2010). Passive cavitation mapping for localization and tracking of bubble dynamics. J. Acoust. Soc. Am..

[151] Haworth K.J., Bader K.B., Rich K.T., Holland C.K., Mast T.D. (2017). Quantitative Frequency-Domain Passive Cavitation Imaging. IEEE Trans. Ultrason. Ferroelectr. Freq. Control.

[152] Coviello C., Kozick R., Choi J., Gyöngy M., Jensen C., Smith P.P., Coussios C.-C. (2015). Passive acoustic mapping utilizing optimal beamforming in ultrasound therapy monitoring. J. Acoust. Soc. Am..

[153] Polichetti M., Varray F., Gilles B., Bera J.C., Nicolas B. (2021). Use of the Cross-Spectral Density Matrix for Enhanced Passive Ultrasound Imaging of Cavitation. IEEE Trans. Ultrason. Ferroelectr. Freq. Control.

[154] Telichko A.V., Lee T., Jakovljevic M., Dahl J.J. (2021). Passive Cavitation Mapping by Cavitation Source Localization From Aperture-Domain Signals-Part I: Theory and Validation Through Simulations. IEEE Trans. Ultrason. Ferroelectr. Freq. Control.

[155] Burgess M.T., Apostolakis I., Konofagou E.E. (2018). Power cavitation-guided blood-brain barrier opening with focused ultrasound and microbubbles. Phys. Med. Biol..

[156] Grundy M., Bau L., Hill C., Paverd C., Mannaris C., Kwan J., Crake C., Coviello C., Coussios C., Carlisle R. (2020). Improved therapeutic antibody delivery to xenograft tumors using cavitation nucleated by gas-entrapping nanoparticles. Nanomedicine.

[157] Sukovich J.R., Macoskey J.J., Lundt J.E., Gerhardson T.I., Hall T.L., Xu Z. (2020). Real-Time Transcranial Histotripsy Treatment Localization and Mapping Using Acoustic Cavitation Emission Feedback. IEEE Trans. Ultrason. Ferroelectr. Freq. Control.

[158] Sun D., Gao W., Hu H., Zhou S. (2022). Why 90% of clinical drug development fails and how to improve it?. Acta Pharm. Sin. B.

[159] Pan E., Bogumil D., Cortessis V., Yu S., Nieva J. (2020). A Systematic Review of the Efficacy of Preclinical Models of Lung Cancer Drugs. Front. Oncol..

[160] Minchinton A.I., Tannock I.F. (2006). Drug penetration in solid tumours. Nat. Rev. Cancer.

[161] Maeda H., Khatami M. (2018). Analyses of repeated failures in cancer therapy for solid tumors: Poor tumor-selective drug delivery, low therapeutic efficacy and unsustainable costs. Clin. Transl. Med..

[162] Sun G., Rong D., Li Z., Sun G., Wu F., Li X., Cao H., Cheng Y., Tang W., Sun Y. (2021). Role of Small Molecule Targeted Compounds in Cancer: Progress, Opportunities, and Challenges. Front. Cell Dev. Biol..

[163] Lee Y.T., Tan Y.J., Oon C.E. (2018). Molecular targeted therapy: Treating cancer with specificity. Eur. J. Pharmacol..

[164] Zhou Z., Li M. (2022). Targeted therapies for cancer. BMC Med..

[165] Haslam A., Kim M.S., Prasad V. (2021). Updated estimates of eligibility for and response to genome-targeted oncology drugs among US cancer patients, 2006–2020. Ann. Oncol..

[166] Benjamin D.J., Xu A., Lythgoe M.P., Prasad V. (2022). Cancer Drug Approvals That Displaced Existing Standard-of-Care Therapies, 2016–2021. JAMA Netw. Open.

[167] Chaudhry M., Lyon P., Coussios C., Carlisle R. (2022). Thermosensitive liposomes: A promising step toward localised chemotherapy. Expert Opin. Drug Deliv..

[168] Lyon P.C., Gray M.D., Mannaris C., Folkes L.K., Stratford M., Campo L., Chung D.Y.F., Scott S., Anderson M., Goldin R. (2018). Safety and feasibility of ultrasound-triggered targeted drug delivery of doxorubicin from thermosensitive liposomes in liver tumours (TARDOX): A single-centre, open-label, phase 1 trial. Lancet Oncol..

[169] Tours University Hospital Targeted Delivery of Chemotherapy with Ultrasound and Microbubbles. https://clinicaltrials.gov/ct2/show/NCT03458975.

[170] University of Oxford (2022). CEeDD: Sonosensitive Particles and Ultrasound to Enhance Drug Delivery in Metastatic Colorectal Cancer. https://www.oncology.ox.ac.uk/clinical-trials/oncology-clinical-trials-office-octo/current-trials/ceedd.

[171] Banerji U., Tiu C.D., Curcean A., Gurung S., O’Leary M., Bush N., Kotopoulis S., Healey A., Kvåle S., McElwaine-Johnn H.H. (2021). Phase I trial of acoustic cluster therapy (ACT) with chemotherapy in patients with liver metastases of gastrointestinal origin (ACTIVATE study). J. Clin. Oncol..

[172] Dillon P. (2022). Focused Ultrasound with Low-Dose Gemcitabine to Augment Immune Control of Early Stage Breast Cancer. Report No. NCT04796220. NCT04796220.

[173] St. Olavs Hospital (2022). Ultrasound-Enhanced Delivery of Chemotherapy to Patients with Liver Metastasis from Breast—And Colorectal Carcinoma—A Randomized Trial. Report No. study/NCT03477019. NCT03477019.

[174] Yuan Y. (2021). A Single-Arm, Single-Center Exploratory Study of the Safety and Effectiveness of High-Intensity Focused Ultrasound Therapy Combined with REGOTORI for Metastatic Colorectal Cancer. Report No. NCT04819516. NCT04819516.

[175] Lee J.Y. (2022). Therapeutic Efficacy and Safety of Concurrent FOLFIRINOX Plus HIFU for Locally Advanced/Borderline Resectable Pancreatic Cancer: A Prospective Single-Center, Single-Arm, Investigator-Initiated, Open-Labeled, Exploratory Clinical Trial. Report No. study/NCT05262452. NCT05262452.

[176] Thomas Jefferson University (2022). Optimizing Ultrasound Enhanced Delivery of Therapeutics. Report No. NCT04821284. NCT04821284.

[177] St. Olavs Hospital (2021). Ultrasound-Enhanced Uptake of Chemotherapy in Patients with Inoperable Pancreatic Ductal Adenocarcinoma—A Randomized Controlled Trial. Report No. NCT04146441. NCT04146441.

[178] Kim A. (2021). A Phase I Study of Lyso-Thermosensitive Liposomal Doxorubicin (LTLD, ThermoDox®) and Magnetic Resonance-Guided High Intensity Focused Ultrasound (MR-HIFU) for Relapsed or Refractory Solid Tumors in Children, Adolescents, and Young Adults. Report No. NCT02536183. NCT02536183.

[179] Gao Y., Du L., Li Q.B., Li Q.B., Zhu L., Yang M., Wang X., Zhao B.B., Ma S. (2022). How physical techniques improve the transdermal permeation of therapeutics: A review. Medicine.

[180] Finnin B.C., Morgan T.M. (1999). Transdermal penetration enhancers: Applications, limitations, and potential. J. Pharm. Sci..

[181] Ita K. (2017). Recent progress in transdermal sonophoresis. Pharm. Dev. Technol..

[182] Paliwal S., Menon G.K., Mitragotri S. (2006). Low-Frequency Sonophoresis: Ultrastructural Basis for Stratum Corneum Permeability Assessed Using Quantum Dots. J. Investig. Dermatol..

[183] Tezel A., Mitragotri S. (2003). Interactions of inertial cavitation bubbles with stratum corneum lipid bilayers during low-frequency sonophoresis. Biophys. J..

[184] Tezel A., Sens A., Tuchscherer J., Mitragotri S. (2001). Frequency dependence of sonophoresis. Pharm. Res..

[185] Wolloch L., Kost J. (2010). The importance of microjet vs shock wave formation in sonophoresis. J. Control. Release.

[186] Bhatnagar S., Schiffter H., Coussios C.-C. (2014). Exploitation of Acoustic Cavitation-Induced Microstreaming to Enhance Molecular Transport. J. Pharm. Sci..

[187] Kushner J., Blankschtein D., Langer R. (2004). Experimental demonstration of the existence of highly permeable localized transport regions in low-frequency sonophoresis. J. Pharm. Sci.

[188] Kushner J., Kim D., So PT C., Blankschtein D., Langer R.S. (2007). Dual-Channel Two-Photon Microscopy Study of Transdermal Transport in Skin Treated with Low-Frequency Ultrasound and a Chemical Enhancer. J. Investig. Dermatol..

[189] Zimon R.L., Lerman G., Elharrar E., Meningher T., Barzilai A., Masalha M., Chintakunta R., Hollander E., Goldbart R., Traitel T. (2018). Ultrasound targeting of Q-starch/miR-197 complexes for topical treatment of psoriasis. J. Control. Release.

[190] Tandara A.A., Mustoe T.A. (2004). Oxygen in Wound Healing—More than a Nutrient. World J. Surg..

[191] Castilla D.M., Liu Z.-J., Velazquez O.C. (2012). Oxygen: Implications for Wound Healing. Adv. Wound Care.

[192] Sen C.K. (2009). Wound healing essentials: Let there be oxygen. Wound Repair Regen..

[193] Cates N.K., Kim P.J. (2022). Topical Oxygen Therapy for Wound Healing: A Critical Evaluation. Surg. Technol. Int..

[194] Oropallo A., Andersen C.A. (2021). Topical Oxygen. StatPearls.

[195] Roe D.F., Gibbins B.L., Ladizinsky D.A. (2010). Topical dissolved oxygen penetrates skin: Model and method. J. Surg. Res..

[196] Castro C.I., Briceno J.C. (2010). Perfluorocarbon-based oxygen carriers: Review of products and trials. Artif. Organs.

[197] Davis S.C., Cazzaniga A.L., Ricotti C., Zalesky P., Hsu L.-C., Creech J., Eaglstein W.H., Mertz P.M. (2007). Topical Oxygen Emulsion: A Novel Wound Therapy. Arch. Dermatol..

[198] Gold M.H., Nestor M.S. (2020). A Supersaturated Oxygen Emulsion for Wound Care and Skin Rejuvenation. J. Drugs Dermatol..

[199] Eisenbrey J.R., Albala L., Kramer M.R., Daroshefski N., Brown D., Liu J.-B., Stanczak M., O’kane P., Forsberg F., Wheatley M.A. (2015). Development of an ultrasound sensitive oxygen carrier for oxygen delivery to hypoxic tissue. Int. J. Pharm..

[200] Ho Y.-J., Hsu H.-C., Wu B.-H., Lin Y.-C., Liao L.-D., Yeh C.-K. (2023). Preventing ischemia-reperfusion injury by acousto-mechanical local oxygen delivery. J. Control. Release.

[201] Wang H., Guo Y., Hu Y., Zhou Y., Chen Y., Huang X., Chen J., Deng Q., Cao S., Hu B. (2023). Ultrasound-controlled nano oxygen carriers enhancing cell viability in 3D GelMA hydrogel for the treatment of myocardial infarction. Int. J. Biol. Macromol..

[202] Philpott M. (2022). Ultrasound-Assisted Ice Nucleation, Fibroblast Migration and Oxygen Delivery in Collagen Scaffolds for Tissue Engineering. Ph.D. Thesis.

[203] Guo R., Xu N., Liu Y., Ling G., Yu J., Zhang P. (2021). Functional ultrasound-triggered phase-shift perfluorocarbon nanodroplets for cancer therapy. Ultrasound Med. Biol..

[204] Xu Z., Hall T.L., Vlaisavljevich E., Lee F.T. (2021). Histotripsy: The first noninvasive, non-ionizing, non-thermal ablation technique based on ultrasound. Int. J. Hyperth. Off. J. Eur. Soc. Hyperthermic Oncol. N. Am. Hyperth. Group.

[205] Unger E.C., Porter T., Culp W., Labell R., Matsunaga T., Zutshi R. (2004). Therapeutic applications of lipid-coated microbubbles. Adv. Drug Deliv. Rev..

[206] Wang J., Memon F., Touma G., Baltsavias S., Jang J.H., Chang C., Rasmussen M.F., Olcott E., Jeffrey R.B., Arbabian A. Capsule ultrasound device: Characterization and testing results. Proceedings of the 2017 IEEE International Ultrasonics Symposium (IUS).

[207] Sharma D., Misba L., Khan A.U. (2019). Antibiotics versus biofilm: An emerging battleground in microbial communities. Antimicrob. Resist. Infect. Control.

[208] Stewart P.S. (2015). Antimicrobial tolerance in biofilms. Microbiol. Spectr..

[209] Hall-Stoodley L., Costerton J.W., Stoodley P. (2004). Bacterial biofilms: From the natural environment to infectious diseases. Nat. Rev. Microbiol..

[210] Mah T.-F. (2012). Biofilm-specific antibiotic resistance. Future Microbiol..

[211] Kaplan J.B. (2009). Therapeutic potential of biofilm-dispersing enzymes. Int. J. Artif. Organs.

[212] Lattwein K.R., Shekhar H., Kouijzer J.J., van Wamel W.J., Holland C.K., Kooiman K. (2020). Sonobactericide: An emerging treatment strategy for bacterial infections. Ultrasound Med. Biol..

[213] Dong Y., Chen S., Wang Z., Peng N., Yu J. (2013). Synergy of ultrasound microbubbles and vancomycin against Staphylococcus epidermidis biofilm. J. Antimicrob. Chemother..

[214] Kouijzer J.J., Lattwein K.R., Beekers I., Langeveld S.A., Leon-Grooters M., Strub J.-M., Oliva E., Mislin G.L., de Jong N., van der Steen A.F. (2021). Vancomycin-decorated microbubbles as a theranostic agent for Staphylococcus aureus biofilms. Int. J. Pharm..

[215] LuTheryn G., Hind C., Campbell C., Crowther A., Wu Q., Keller S.B., Glynne-Jones P., Sutton J.M., Webb J.S., Gray M. (2022). Bactericidal and anti-biofilm effects of uncharged and cationic ultrasound-responsive nitric oxide microbubbles on Pseudomonas aeruginosa biofilms. Front. Cell. Infect. Microbiol..

[216] Plazonic F., LuTheryn G., Hind C., Clifford M., Gray M., Stride E., Glynne-Jones P., Hill M., Sutton J.M., Carugo D. (2022). Bactericidal effect of ultrasound-responsive microbubbles and sub-inhibitory gentamicin against Pseudomonas aeruginosa biofilms on substrates with differing acoustic impedance. Ultrasound Med. Biol..

[217] Lattwein K.R., Shekhar H., van Wamel W.J.B., Gonzalez T., Herr A.B., Holland C.K., Kooiman K. (2018). An in vitro proof-of-principle study of sonobactericide. Sci. Rep..

[218] Horsley H., Owen J., Browning R., Carugo D., Malone-Lee J., Stride E., Rohn J. (2019). Ultrasound-activated microbubbles as a novel intracellular drug delivery system for urinary tract infection. J. Control. Release.

[219] Dong Y., Li J., Li P., Yu J. (2018). Ultrasound microbubbles enhance the activity of vancomycin against Staphylococcus epidermidis biofilms in vivo. J. Ultrasound Med..

[220] Ronan E., Edjiu N., Kroukamp O., Wolfaardt G., Karshafian R. (2016). USMB-induced synergistic enhancement of aminoglycoside antibiotics in biofilms. Ultrasonics.

[221] He N., Hu J., Liu H., Zhu T., Huang B., Wang X., Wu Y., Wang W., Qu D. (2011). Enhancement of vancomycin activity against biofilms by using ultrasound-targeted microbubble destruction. Antimicrob. Agents Chemother..

[222] Chua S.L., Liu Y., Yam J.K.H., Chen Y., Vejborg R.M., Tan B.G.C., Kjelleberg S., Tolker-Nielsen T., Givskov M., Yang L. (2014). Dispersed cells represent a distinct stage in the transition from bacterial biofilm to planktonic lifestyles. Nat. Commun..

[223] Walters M.C., Roe F., Bugnicourt A., Franklin M.J., Stewart P.S. (2003). Contributions of antibiotic penetration, oxygen limitation, and low metabolic activity to tolerance of Pseudomonas aeruginosa biofilms to ciprofloxacin and tobramycin. Antimicrob. Agents Chemother..

[224] Anderl J.N., Zahller J., Roe F., Stewart P.S. (2003). Role of nutrient limitation and stationary-phase existence in Klebsiella pneumoniae biofilm resistance to ampicillin and ciprofloxacin. Antimicrob. Agents Chemother..

[225] Barna J., Williams D. (1984). The structure and mode of action of glycopeptide antibiotics of the vancomycin group. Annu. Rev. Microbiol..

[226] Barraud N., JKelso M., ARice S., Kjelleberg S. (2015). Nitric oxide: A key mediator of biofilm dispersal with applications in infectious diseases. Curr. Pharm. Des..

[227] Kelm M. (1999). Nitric oxide metabolism and breakdown. Biochim. Biophys. Acta (BBA)-Bioenerg..

[228] Kadry H., Noorani B., Cucullo L. (2020). A blood–brain barrier overview on structure, function, impairment, and biomarkers of integrity. Fluids Barriers CNS.

[229] Demeule M., Régina A., Jodoin J., Laplante A., Dagenais C., Berthelet F., Moghrabi A., Béliveau R. (2002). Drug transport to the brain: Key roles for the efflux pump P-glycoprotein in the blood–brain barrier. Vasc. Pharmacol..

[230] Ben-Zvi A., Lacoste B., Kur E., Andreone B.J., Mayshar Y., Yan H., Gu C. (2014). Mfsd2a is critical for the formation and function of the blood–brain barrier. Nature.

[231] Reese T., Karnovsky M.J. (1967). Fine structural localization of a blood-brain barrier to exogenous peroxidase. J. Cell Biol..

[232] Ballabh P., Braun A., Nedergaard M. (2004). The blood–brain barrier: An overview: Structure, regulation, and clinical implications. Neurobiol. Dis..

[233] Abbott N.J., Patabendige A.A., Dolman D.E., Yusof S.R., Begley D.J. (2010). Structure and function of the blood–brain barrier. Neurobiol. Dis..

[234] Pardridge W.M. (2005). The blood-brain barrier: Bottleneck in brain drug development. NeuroRx.

[235] Kinoshita M., McDannold N., Jolesz F.A., Hynynen K. (2006). Targeted delivery of antibodies through the blood–brain barrier by MRI-guided focused ultrasound. Biochem. Biophys. Res. Commun..

[236] Treat L.H., McDannold N., Zhang Y., Vykhodtseva N., Hynynen K. (2012). Improved anti-tumor effect of liposomal doxorubicin after targeted blood-brain barrier disruption by MRI-guided focused ultrasound in rat glioma. Ultrasound Med. Biol..

[237] Burgess A., Huang Y., Querbes W., Sah D.W., Hynynen K. (2012). Focused ultrasound for targeted delivery of siRNA and efficient knockdown of Htt expression. J. Control. Release.

[238] Abbott N.J. (2000). Inflammatory mediators and modulation of blood–brain barrier permeability. Cell. Mol. Neurobiol..

[239] McDannold N., Vykhodtseva N., Raymond S., Jolesz F.A., Hynynen K. (2005). MRI-guided targeted blood-brain barrier disruption with focused ultrasound: Histological findings in rabbits. Ultrasound Med. Biol..

[240] Durán-Lobato M., Niu Z., Alonso M.J. (2020). Oral Delivery of Biologics for Precision Medicine. Adv. Mater..

[241] Smart A.L., Gaisford S., Basit A.W. (2014). Oral peptide and protein delivery: Intestinal obstacles and commercial prospects. Expert Opin. Drug Deliv..

[242] Varum FJ O., Hatton G.B., Basit A.W. (2013). Food, physiology and drug delivery. Int. J. Pharm..

[243] Schoellhammer C.M., Langer R., Traverso G. (2016). Of microneedles and ultrasound: Physical modes of gastrointestinal macromolecule delivery. Tissue Barriers.

[244] Newman M.K., Kill M., Frampton G. (1958). Effects of ultrasound alone and combined with hydrocortisone injections by needle or hypo-spray. Am. J. Phys. Med..

[245] Polat B.E., Blankschtein D., Langer R. (2010). Low-frequency sonophoresis: Application to the transdermal delivery of macromolecules and hydrophilic drugs. Expert Opin. Drug Deliv..

[246] Polat B.E., Hart D., Langer R., Blankschtein D. (2011). Ultrasound-mediated transdermal drug delivery: Mechanisms, scope, and emerging trends. J. Control. Release.

[247] Prausnitz M.R., Langer R. (2008). Transdermal drug delivery. Nat. Biotechnol..

[248] Mitragotri S., Blankschtein D., Langer R. (1995). Ultrasound-Mediated Transdermal Protein Delivery. Science.

[249] Schoellhammer C.M., Polat B.E., Mendenhall J., Maa R., Jones B., Hart D.P., Langer R., Blankschtein D. (2012). Rapid skin permeabilization by the simultaneous application of dual-frequency, high-intensity ultrasound. J. Control. Release.

[250] Schoellhammer C.M., Srinivasan S., Barman R., Mo S.H., Polat B.E., Langer R., Blankschtein D. (2015). Applicability and safety of dual-frequency ultrasonic treatment for the transdermal delivery of drugs. J. Control. Release.

[251] Bawiec C.R., Sunny Y., Nguyen A.T., Samuels J.A., Weingarten M.S., Zubkov L.A., Lewin P.A. (2013). Finite element static displacement optimization of 20-100 kHz flexural transducers for fully portable ultrasound applicator. Ultrasonics.

[252] Schoellhammer C.M., Schroeder A., Maa R., Lauwers G.Y., Swiston A., Zervas M., Barman R., DiCiccio A.M., Brugge W.R., Anderson D.G. (2015). Ultrasound-mediated gastrointestinal drug delivery. Sci. Transl. Med..

[253] Schoellhammer C.M., Lauwers G.Y., Goettel J.A., Oberli M.A., Cleveland C., Park J.Y., Minahan D., Chen Y., Anderson D.G., Jaklenec A. (2017). Ultrasound-Mediated Delivery of RNA to Colonic Mucosa of Live Mice. Gastroenterology.

[254] Schoellhammer C.M., Chen Y., Cleveland C., Minahan D., Bensel T., Park J.Y., Saxton S., Lee Y.-A.L., Booth L., Langer R. (2017). Defining optimal permeant characteristics for ultrasound-mediated gastrointestinal delivery. J. Control. Release.

[255] France M.M., del Rio T., Travers H., Raftery E., Xu K., Langer R., Traverso G., Lennerz J.K., Schoellhammer C.M. (2019). Ultra-rapid drug delivery in the oral cavity using ultrasound. J. Control. Release.

[256] France M.M., del Rio T., Travers H., Raftery E., Langer R., Traverso G., Schoellhammer C.M. (2022). Platform for the Delivery of Unformulated RNA In Vivo. J. Pharm. Sci..

[257] Stewart F., Verbeni A., Qiu Y., Cox B.F., Vorstius J., Newton I.P., Huang Z., Menciassi A., Näthke I., Cochran S. (2018). A Prototype Therapeutic Capsule Endoscope for Ultrasound-Mediated Targeted Drug Delivery. J. Med. Robot. Res..

[258] Stewart F.R., Qiu Y., Lay H.S., Newton I.P., Cox B.F., Al-Rawhani M.A., Beeley J., Liu Y., Huang Z., Cumming D.R.S. (2017). Acoustic Sensing and Ultrasonic Drug Delivery in Multimodal Theranostic Capsule Endoscopy. Sensors.

[259] Stewart F., Cummins G., Turcanu M.V., Cox B.F., Prescott A., Clutton E., Newton I.P., Desmulliez M.P.Y., Thanou M., Mulvana H. (2021). Ultrasound mediated delivery of quantum dots from a proof of concept capsule endoscope to the gastrointestinal wall. Sci. Rep..

[260] Fix S.M., Koppolu B.P., Novell A., Hopkins J., Kierski T.M., Zaharoff D.A., Dayton P.A., Papadopoulou V. (2019). Ultrasound-Stimulated Phase-Change Contrast Agents for Transepithelial Delivery of Macromolecules, Toward Gastrointestinal Drug Delivery. Ultrasound Med. Biol..

